# Supercritical CO_2_ Antisolvent-Micronised Naringin and Naringenin Alleviate Paclitaxel-Induced Pain Syndrome

**DOI:** 10.3390/pharmaceutics18060747

**Published:** 2026-06-17

**Authors:** Gabriela Adriany Lisboa Zilli, Samara Cristina Mazon, Patricia Viera de Oliveira, Felipe Zaniol, Eulália Lopes da Silva Barros, Ângela Maria Lodi, Chaiane Lunelli Saretto, Hemyly Cardoso, Ana Lúcia Anversa Segatto, Sara Marchesan Oliveira, J. Vladimir Oliveira, Indiara Brusco

**Affiliations:** 1 Graduate Program in Environmental Sciences, Community University of Chapecó Region—Unochapecó, Chapecó 89809-900, SC, Brazilsamaramazon2507@gmail.com (S.C.M.);; 2 Behavioural and Neurochemistry Pharmacology of Pain Research Group, Community University of Chapecó Region—Unochapecó, Chapecó 89809-900, SC, Brazil; 3 Department of Chemical and Food Engineering, Federal University of Santa Catarina—UFSC, Florianópolis 88040-900, SC, Brazilfelipezaniol2@gmail.com (F.Z.); barroseulalialopes@gmail.com (E.L.d.S.B.); 4 Graduate Program in Biological Sciences: Toxicological Biochemistry, Centre of Natural and Exact Sciences, Federal University of Santa Maria—UFSM, Santa Maria 97105-900, RS, Brazil; analuciasegatto@gmail.com (A.L.A.S.);

**Keywords:** nociception, flavonoids, capsaicin, neuropathy, allodynia

## Abstract

**Background**/**Objectives**: Paclitaxel is a chemotherapy drug used to treat various tumours, but its use is often limited by an acute and chronic pain syndrome that is poorly managed. Naringin and its aglycone, naringenin, exhibit antioxidant, antitumour, anti-inflammatory, and antinociceptive effects, making them potential alternative treatments. However, their low water solubility limits their oral bioavailability in humans. Micronisation in a supercritical medium reduces particle size and enhances the dissolution of compounds, offering a possible solution. In this study, we investigated whether micronising naringin and naringenin via supercritical technology could improve their dissolution and oral efficacy against paclitaxel-induced pain syndrome. **Methods**: Micronisation was performed using supercritical CO_2_. Molecular docking was used to analyse the binding of naringin and naringenin to TRPV1, a key target for pain relief. Swiss mice were used in capsaicin (TRPV1 agonist)-induced nociception and paclitaxel-caused acute and chronic pain models. We assessed mechanical, cold, and heat sensitivity, potential adverse effects, and TRPV1 mRNA expression. **Results**: Micronisation improved the apparent dissolution profile of molecules. Docking results showed that naringin and naringenin bind to TRPV1. Both micronised compounds reduced capsaicin-induced nociception without affecting locomotion or body temperature. Micronised naringin and naringenin alleviated mechanical and cold allodynia, as well as thermal hyperalgesia in both acute and chronic paclitaxel-induced pain, outperforming their conventional forms. They also downregulated TRPV1 mRNA expression in the mice’s sciatic nerve. **Conclusions**: Taken together, these results show that supercritical micronisation improved the apparent dissolution and oral antinociceptive efficacy of naringin and naringenin, emphasising their potential as promising alternatives for managing paclitaxel-induced pain, with TRPV1 being a probable contributor to the observed antinociceptive effects.

## 1. Introduction

The latest data from the Global Cancer Observatory (2020–2022) estimates 20 million new cancer cases and a prevalence of 50.5 million cases diagnosed in the last five years [1,2]. It is expected that by 2050, there will be 35 million new cases worldwide, representing a 77% increase compared to 2022 [1]. The most common types of cancer include lung, breast, colorectal, prostate, and stomach cancers [1,2]. Among the available therapeutic options, paclitaxel is a widely used chemotherapy drug for cancer treatment, including the most common cancer types [2,3]. Despite its effectiveness, paclitaxel can cause neurotoxicity, which manifests as a painful syndrome characterised by acute and neuropathic pain that may limit its use and reduce treatment adherence by patients [4,5,6,7,8].

Chemotherapy-induced neuropathic pain affects approximately 65–80% of patients undergoing chemotherapy, including those receiving paclitaxel, and may last for months or even years after treatment ends [4,9,10,11]. Furthermore, paclitaxel-induced acute pain usually begins within the first hour of infusion and can be severe during the initial days of treatment, affecting up to 58% of patients [12,13]. This pain syndrome is characterised by numbness or discomfort in the hands and feet, spontaneous pain, mechanical and cold allodynia (pain from non-painful stimuli), and hyperalgesia (increased sensitivity to painful stimuli) [5,7,9,11,14,15].

Several mechanisms have been proposed to be involved in paclitaxel-induced pain syndrome, including tubulin stabilisation in axons, increased oxidative stress, the Transient Receptor Potential Vanilloid 1 (TRPV1) ionic channel, and kinin receptors, among others [7,16,17,18,19,20]. However, there are still no specific treatments recommended for alleviating chemotherapy-induced pain, and duloxetine is the only drug with substantial evidence of efficacy, although its benefit remains limited [5]. Therefore, new therapeutic strategies for paclitaxel-induced pain syndrome attenuation are needed.

Natural products are a valuable source of compounds for pain management, including targeting ion channels [21,22]. Flavonoids naringin and its aglycone naringenin, mainly found in citrus fruits, have attracted interest due to their antinociceptive, antioxidant, anti-inflammatory, and anti-apoptotic properties [23,24,25]. These compounds have shown efficacy in preclinical models of arthritic, cancer-induced, and diabetic neuropathic pain [23,25,26,27]. Moreover, clinical trials show that naringin and naringenin are relatively safe and non-toxic [24].

Recent studies showed that a naringenin-derived flavanone exhibited superior antinociceptive effects compared to naringenin itself when administered intrathecally in a paclitaxel-induced pain model, likely by inhibiting P2X purinoceptor 7 (P2X7) function and calcitonin gene-related peptide (CGRP) production [28]. Similarly, intraperitoneal administration of naringenin reduced paclitaxel-induced pain and improved antitumour activity by downregulating CGRP signalling [29]. However, both studies used non-oral routes, limiting their clinical applicability. Relevantly, naringin effects on the acute and chronic paclitaxel-induced pain syndrome have not been evaluated until now.

Notably, TRPV1 channels are involved in CGRP release, which has been implicated in chemotherapy-induced pain and migraine [30,31]. The relation between TRPV1 and CGRP has also been demonstrated in the paclitaxel-induced pain model [32,33]. Additionally, naringin presented TRPV1 antagonist-like properties, suggesting a potential analgesic effect through this channel [34]. TRPV1 channels are expressed in nociceptive pathways and are considered targets for pain-relief studies [35]. Therefore, we investigated whether TRPV1 could also be contributing to the antinociceptive effects of naringenin and naringin in paclitaxel-induced pain.

Although preclinical studies show promising pharmacological potential for naringin and naringenin, their low water solubility restricts oral bioavailability, thereby limiting their clinical application [24]. In this sense, supercritical CO_2_-assisted micronisation has emerged as an efficient strategy to enhance dissolution behaviour and bioavailability by reducing particle size and increasing specific surface area [36,37]. Studies have confirmed the enhanced dissolution behaviour and pharmacological properties of micronised forms of naringin and naringenin [36,38]. Therefore, we investigated the oral antinociceptive effects of micronised naringin and naringenin in a paclitaxel-induced pain syndrome model, and the possible contribution of TRPV1 to these effects.

## 2. Materials and Methods

### 2.1. Reagents and Treatments

All reagents, unless otherwise specified, were purchased from Sigma-Aldrich Chemical Company (St. Louis, MO, USA). Ethanol (99.5%) and acetone (99.5%) (Êxodo Científico; São Paulo, Brazil) were used as solvents for the precipitation of naringenin and naringin, respectively. Liquid CO_2_ from White Martins S.A. (São Paulo, Brazil) was used as a supercritical fluid. Naringin and naringenin were dissolved in distilled water containing 5% Tween 80 and 1% DMSO and administered orally by gavage (p.o.). Paclitaxel (6 mg/mL in Cremophor EL and dehydrated ethanol, 1:1 ratio and final concentration 0.83%/0.83%) was obtained from Glenmark (Buenos Aires, Argentina), diluted in saline (0.9% NaCl), and administered intraperitoneally (i.p.). A stock solution of capsaicin was prepared in ethanol (4% *v*/*v*), Tween (4% *v*/*v*), and PBS (92% *v*/*v*). From this stock, capsaicin was dissolved in phosphate-buffered saline (PBS, 0.9% NaCl) and administered via the intraplantar route (i.pl.) at 1 nmol/paw. All control groups (vehicles) received the respective vehicles in which the treatments were dissolved. The doses used in the nociception models followed previous studies [16,39], including doses of naringin and naringenin on a logarithmic scale [23,36,40]. Micronisation, particle characterisation, and dissolution testing were performed by the Laboratory of Thermodynamics and Supercritical Technology (LATESC) at the Federal University of Santa Catarina (UFSC).

### 2.2. Gas Anti-Solvent Micronisation (GAS)

The Gas Anti-Solvent (GAS) method was performed following the procedures described in previous studies [41,42]. A 17.5 mg.mL^−1^ solution of naringenin (ethanol) and a 10 mg mL^−1^ solution of naringin (acetone) were prepared [38]. The solution was passed through a 0.45 μm PTFE (polytetrafluoroethylene) filter. It was placed in a chamber (600 mL with an internal diameter of 8 cm and a height of 12 cm), and CO_2_ was pumped in using a syringe pump at a rate of 10 mL.min^−1^ for naringenin and 15 mL.min^−1^ for naringin until reaching the desired operating pressure of 80 bar, under a constant temperature of 35 °C (naringenin) and 45 °C (naringin) maintained by a thermostatic bath. The pressure and temperature settings were based on previous studies [36,38,43,44]. Once the pressure was reached, the antisolvent flow was stopped, and the chamber was maintained under magnetic stirring for 10 min. After this period, the washing step began with the antisolvent flow at 10 mL.min^−1^ for naringenin and 15 mL.min^−1^ for naringin, maintaining the isobaric pressure for 60 min. Finally, the micronised particles were collected for subsequent characterisation and in vivo evaluation. The same characterised particles were analysed in vivo. Residual solvent content was not measured for the specific batches used in this study, which is a limitation. However, previous studies using the same GAS conditions for these particles reported residual solvent levels below the quantification limit [36]. The study used characterised material from the prepared batch/pool used for in vivo testing; independent batch-to-batch reproducibility was not evaluated. The micronised particles were stored at −20 °C in sealed containers protected from light and moisture and were used in the in vivo experiments approximately 6–7 weeks after micronisation. Future studies should assess physicochemical stability during storage, especially for the partially amorphous micronised naringin.

### 2.3. Characterisation of Particles

Particle morphology, mean diameter, and size distribution were determined by scanning electron microscopy (SEM) using a JEOL JSM-6390LV microscope, with image analysis performed using ImageJ 1.54g and data processing carried out in OriginPro 2016 software (OriginLab Corporation, Northampton, MA, USA), considering approximately 300 individual particle measurements obtained from the analysed pooled sample for particle-size distribution analysis [36,45,46]. X-ray diffraction (XRD) analyses were performed using a Rigaku MiniFlex600 benchtop diffractometer (Rigaku Corporation, Tokyo, Japan). The measurement range (2θ) was 5–35°, with a step size of 0.02° and a scan speed of 6°/s. Fourier transform infrared (FTIR) spectroscopy was carried out using an Agilent Cary 600 spectrometer (Agilent Technologies, Santa Clara, CA, USA) equipped with an attenuated total reflectance (ATR) accessory with a ZnSe crystal, over the spectral range of 4000–500 cm^−1^ at a resolution of 2 cm^−1^. The thermal behaviour was evaluated by differential scanning calorimetry (DSC) using a PerkinElmer Jade DSC instrument (PerkinElmer, Shelton, CT, USA). Samples were placed in hermetically sealed aluminium crucibles and subjected to a single heating cycle from 0 to 300 °C under a dynamic nitrogen atmosphere (20 mL min^−1^) at a heating rate of 10 °C·min^−1^ [36,38,46].

### 2.4. Evaluation of the Dissolution Profile

The dissolution behaviour of naringenin and naringin, in both their conventional and micronised forms, was evaluated using a modified version of the method described by Cheng et al. (2016) [47]. An excess amount of solid was used to conduct the assay under non-sink conditions, allowing comparison of the apparent dissolution behaviour of conventional and micronised particles rather than the determination of true sink dissolution kinetics. About 10 mg of each sample was added to 100 mL of two dissolution media: an acidic medium (0.1 M HCl, pH 1.2) and a phosphate-buffered saline (PBS, pH 6.8) solution. Experiments were carried out under constant agitation (100 rpm) at 37 ± 0.5 °C with a Dubnoff-type shaker (Marconi, Piracicaba, São Paulo, Brazil). At specified time points (5, 10, 15, 20, 30, 40, 50, 60, 90, 120, and 180 min), 2 mL aliquots were withdrawn without replacement of fresh medium, immediately filtered through a 0.22 μm PTFE syringe filter, and analyzed using a UV-Vis spectrophotometer (model T90+, PG Instruments, Leicestershire, UK) at 288 nm for naringenin and 285 nm for naringin. All tests were performed in triplicate for both media, and the results were expressed as solute concentration (μg·mL^−1^) over time. Because aliquots were withdrawn without replacing the fresh medium, the results are presented as apparent concentration profiles from non-replacement sampling. Therefore, the dissolution assay was intended only to compare the relative performance of micronised and commercial naringin under identical experimental conditions and should not be interpreted as an absolute determination of dissolution or solubility. This interpretation should also be considered in light of the experimental design, including the use of UV-Vis calibration curves prepared in ethanol rather than in the dissolution media, the cumulative withdrawal of approximately 22% of the initial dissolution volume, and the occurrence of some measurements above the upper calibration limit. These measurements were retained solely for the qualitative and comparative interpretation of the apparent concentration profiles and should be considered semi-quantitative estimates rather than fully validated quantitative results. Consequently, the reported values should be regarded as apparent concentration profiles suitable for comparative evaluation of the tested formulations. Further details are provided in the Supplementary Material.

### 2.5. In Silico Assay—Molecular Docking

Molecular docking was performed to assess the potential of naringenin and naringin to interact with TRPV1 channels. The structures of naringenin (CID: 439246) and naringin (CID: 442428) were obtained from PubChem (https://pubchem.ncbi.nlm.nih.gov/, accessed 15 September 2025) and optimised using Avogadro software 2.0.0 [48,49]. The structure of the TRPV1 channel (PDB ID:3J5P) was obtained from the Protein Data Bank [50]. The details of the simulation box include the Cartesian coordinates for naringenin and naringin (X = 40 Å, Y = 40 Å, Z = 40 Å) and the grid box centre coordinates for TRPV1 (X = −29 Å, Y= 10 Å, Z = −13 Å), which were defined using the CB-DOCK2 tool [51]. Molecular docking was performed using AMDock version 1.5.2 [52] and AutoDock Vina 1.2.1 [53], following the methodology described by [54]. The results of molecular docking were visualised with PyMOL version 4.6.0 and BIOVIA Discovery Studio Visualizer 64 programs [55,56].

### 2.6. Animals

Adult male Swiss mice (25–30 g) were used in the experiments. The animals were obtained from the Central Animal Facility of the Community University of the Chapecó Region (Unochapecó), Santa Catarina, Brazil. They were housed in polypropylene boxes with identification labels and maintained at a controlled temperature (22–23 °C), with a 12 h light/dark cycle and free access to food and water. All behavioural tests were conducted between 8:00 a.m. and 5:00 p.m. Additionally, the animals were acclimated to the experimental room, the procedures, and the research team conducting the experiments. Experimental protocols followed ethical guidelines established for investigations of experimental pain in conscious animals [57], and the number of animals and intensity of all noxious stimuli used were the minimum required to reveal the effects of the treatments. A total of 6 animals per group was established based on previous studies [16,58,59]. Some groups had n = 5 due to the removal of outlier animals, failure to record an open-field video, and limited availability of animals from the breeding facility (n total = 215 animals). The exact outliers are indicated in the data availability document (File S1). No randomisation programme was used, which is a limitation. However, animals were allocated based on baseline thresholds for mechanical and cold allodynia, measured before and after paclitaxel administration, to ensure all animals started from the same baseline. For other experiments, animals were allocated to ensure a homogeneous body-weight distribution prior to treatments, thereby guaranteeing the use of the same micronised batches across all protocols. For blinding, different evaluators administered treatments and performed the analyses. The experimental protocols were approved by the Institutional Animal Care and Use Committee of Unochapecó under approval number CEUA/002/2023 (18 April 2023). All ethical aspects, including humane endpoints, were conducted in accordance with national legislation (guidelines of the Brazilian Council of Animal Experimentation Control—CONCEA).

### 2.7. Capsaicin-Induced Nociception (Screening Test)

The antinociceptive effects of micronised naringin and naringenin were evaluated using a dose–response curve (30, 100, and 300 mg/kg, p.o.) to capsaicin-induced nociception, and the effects were compared with those of their conventional forms (non-micronised). First, animals were individually placed in transparent glass cylinders (20 cm in diameter) and acclimatised for 20 min. Next, mice were treated with vehicle (10 mL/kg, p.o.) or with different doses of conventional or micronised naringin and naringenin. After 1 h of treatment, animals received an intraplantar (i.pl.) vehicle (PBS, 20 μL/paw) or capsaicin (1 nmol/paw) [39]. Nociceptive behaviour, measured by paw licking, was assessed immediately for 5 min, and results were expressed as paw-licking time in seconds [39]. The intermediate dose identified in this test (100 mg/kg, p.o.) was used in the paclitaxel-induced pain syndrome model.

### 2.8. Evaluation of Adverse Effects

Potential adverse effects of changes in body temperature, sedation, and loss of motor coordination were assessed 1 h after treatment with vehicle (10 mL/kg, p.o.) or different doses of conventional or micronised naringin and naringenin (30, 100, and 300 mg/kg, p.o.). Locomotor activity was measured to assess possible sedative or altered-movement effects. Spontaneous locomotor activity was evaluated using the open field test in a box divided into 12 quadrants (33 × 33 cm). The distance travelled and speed were recorded over 5 min. The videos were analysed using ANY-Maze® 7.65 software (Stoelting Co., Wood Dale, IL, USA), with the data exported for statistical analysis. Additionally, forced locomotion was tested using the rotarod apparatus (3.7 cm diameter, 16 rpm). Animals were trained on the device 24 h before testing, and on the test day, the number of falls was recorded for 4 min. Rectal temperature was measured with a digital thermometer, and results were expressed as the difference in temperature (Δ °C) between baseline and post-treatment measurements [58].

### 2.9. mRNA TRPV1 Expression in Paclitaxel-Induced Pain

To verify the role of TRPV1 channels in paclitaxel-related pain, a neuropathic pain model was induced in mice [16,58], and TRPV1 gene expression was analysed by quantitative real-time PCR (qRT-PCR) [60,61]. Twenty-one days after the first paclitaxel injection (1 + 1 + 1 + 1 mg/kg, i.p.) or vehicle (10 mL/kg, i.p.), the mice were treated with vehicle (10 mL/kg, p.o.) or with micronised or conventional forms of naringin and naringenin (100 mg/kg, p.o.), and sciatic nerve samples were collected one hour after treatment.

Total RNA was isolated from samples using the PureLink RNA Mini Kit (Thermo Fisher Scientific, Waltham, MA, USA) according to the manufacturer’s recommendations and quantified using the Qubit RNA HS Assay Kit (Thermo Fisher Scientific). The cDNA was synthesised using the High-Capacity cDNA Reverse Transcription Kit (Thermo Fisher Scientific) and quantified with the Qubit dsDNA HS Assay Kit (Thermo Fisher Scientific). The samples were prepared to a final concentration of 5 ng/μL, and qRT-PCR was performed using the PowerUp SYBR Green Master Mix (Thermo Fisher Scientific) following the manufacturer’s instructions. As internal controls to normalise gene expression, β-actin (NM 001101) (primer sequence 5′–3′ GACTCATCGTACTCCTGCTTG-Reverse and GATTACTGCTCTGGCTCCTAG-Forward) and Gapdh (NM 001394060.1) (primer sequence 5′–3′ TTCAGCTCTGGGATGACCTT-Reverse and TGCCACTCAGAAGACTGTGG-Forward) were used [60,62]. The geometric mean of β-actin and GAPDH was used for normalisation, reducing variability in individual reference genes and yielding more stable and reliable expression measurements. This approach was supported by analysis with the RefFinder program, which integrates the geNorm, NormFinder, BestKeeper, and comparative ΔCt algorithms and identified the combined expression of these two genes as more stable than either gene alone (Figure S1). The gene of interest was TRPV1 (NM 031982) (primer sequence 5′–3′ GGCATTGACAAACTGCTTCAGGCT-Reverse and GCACAATGGGCAGAATGACACCAT-Forward) [63].

Each reaction contained 10 ng of cDNA and 0.5 mM of each primer in a final volume of 10 μL, and each sample was analysed in triplicate. The PCR cycling conditions were as follows: 50 °C for 2 min, 95 °C for 10 min, then 40 cycles at 95 °C for 15 s and 60 °C for 1 min. Dissociation was performed at 95 °C (1.6 °C/s) for 15 s, followed by 60 °C (1.6 °C/s) for 1 min, and finally 95 °C (1.6 °C/s) for 15 s. qRT-PCR was performed on the QuantStudio 3 (Thermo Fisher Scientific). Relative gene expression levels were calculated using the RQ = 2^−ΔΔCt^ method [64]. Since TRPV1 expression was reduced by naringin and naringenin, we investigated whether these molecules, in their conventional or micronised forms, could alleviate nociceptive behaviours in the paclitaxel-induced neuropathic pain model and also in the acute pain model.

### 2.10. Mechanical Sensitivity

A reduction in the mechanical paw withdrawal threshold (PWT) compared to baseline was considered evidence of mechanical allodynia. The PWT was measured using the up-and-down method described by Chaplan et al. (1994) [65] and adapted for mice [16,58]. Mice were initially acclimated in individual acrylic chambers (7 × 9 × 11 cm) placed on an elevated wire mesh platform to access the plantar surface of the hind paws. von Frey filaments of increasing stiffness (0.02–10.0 g) were applied perpendicularly to the plantar surface with enough force to bend the filament. If there was no paw withdrawal within 5 s, a filament with a higher force was used. A positive response (paw withdrawal) prompted the use of a weaker filament. This process continued until six measurements were taken or until four consecutive positive or negative responses were observed. The 50% mechanical PWT was expressed in grams (g) and calculated as previously described [65,66], using a δ value of 0.469.

### 2.11. Cold Sensitivity

Cold allodynia was defined as an increased sensitivity to cold compared to baseline (B1). Cold sensitivity was assessed using the acetone drop method, where a drop of acetone (20 µL) was applied three times to each right hind paw. Cumulative scores were calculated as follows: 0 = no response; 1 = brief withdrawal, shaking, or stamping of the paw; 2 = prolonged withdrawal or repeated shaking of the paw; 3 = repeated shaking accompanied by licking of the ventral surface. The results were expressed as the total of these scores [67].

#### Evaluation of the Effect of Conventional or Micronised Naringin and Naringenin on Paclitaxel-Induced Mechanical and Cold Allodynia

First, the animals’ baseline behaviour (baseline 1; B1) was assessed in response to mechanical stimulation with von Frey filaments and to cold stimulation with a drop of acetone. Then, the animals received a single dose of paclitaxel (1 mg/kg, i.p.) (acute pain protocol) or repeated doses on alternate days (1 + 1 + 1 + 1 mg/kg, i.p.) (neuropathic pain protocol) [16,58]. The same behaviours were reassessed 24 h after acute pain induction or 21 days after the first repeated paclitaxel administration, defining baseline 2 (B2). Next, animals were treated with vehicle (10 mL/kg, p.o.) or conventional or micronised naringin and naringenin (100 mg/kg, p.o.). Mechanical PWT was measured at 0.5, 1, 2, 4, 6, and 8 h after treatment, and cold sensitivity was evaluated at 1, 2, 4, 6, and 8 h.

### 2.12. Thermal Sensitivity

Thermal hyperalgesia to heat was defined as a shorter paw withdrawal latency compared to the vehicle group and was assessed using the hot plate test, as described previously [68]. On the test day, animals were placed on the hot plate apparatus (EFF 361—Insight Equipment) maintained at 50 ± 1 °C, and the response latency, measured as the time until the animal shakes, licks its hind paws, or jumps, was recorded as the response latency index. A 30 s cutoff time was used to prevent tissue damage. Results are expressed as response latency in seconds (s). To reduce the number of animals, the same animals were used in both the acute and chronic models and remained in the same treatment groups. This is possible because a single exposure to the hot plate and acute oral treatment (day 2) do not interfere with chronic responses, which are evaluated at 21 days.

#### Effect of Conventional or Micronised Naringin and Naringenin on Paclitaxel-Induced Thermal Hyperalgesia

Firstly, the animals received a single administration of paclitaxel (1 mg/kg, i.p.) (acute pain protocol) or repeated administrations of paclitaxel on alternate days (1 + 1 + 1 + 1 mg/kg, i.p.) (neuropathic pain protocol) [16,58]. A control group received a single or repeated administration of vehicle (10 mL/kg, i.p.). At 24 h after acute pain induction or 21 days after the first repeated paclitaxel administration, animals were treated with vehicle (10 mL/kg, p.o.) or conventional or micronised naringin and naringenin (100 mg/kg, p.o.). Latency to respond to the hot plate test was evaluated 1 h after treatment.

### 2.13. Statistical Analysis

Statistical analyses of the in vivo and ex vivo tests were conducted using GraphPad Prism 8.0 software (GraphPad, San Diego, CA, USA). The data were assessed for normality with the Shapiro–Wilk test. Mechanical sensitivity results were log-transformed to achieve normal distribution. Parametric results are shown as the mean ± standard error of the mean (SEM), while non-parametric data are presented as the median and interquartile range. Parametric data were analysed with one-way or two-way ANOVA followed by Bonferroni’s post hoc test. Non-parametric data were analysed with the Kruskal–Wallis test followed by Dunn’s test. Cold allodynia was evaluated by a mixed-effects model (REML) followed by Bonferroni’s post hoc test. *p*-values below 0.05 were considered statistically significant. Area under the curve (AUC) values were calculated for mechanical PWT and cold sensitivity over time (0.5–6 or 8 h; 1–8 h). Outliers were defined using Grubb’s statistical test. The % inhibition of mechanical and cold allodynia x = [(treatment at 1 h − vehicle at 1 h)/(vehicle at basal − vehicle at 1 h)] × 100, and % inhibition for other tests: x = [(treatment − algogenic)/(vehicle − algogenic)] × 100. The % inhibition is expressed as the mean ± SEM. Paclitaxel or capsaicin is considered algogenic.

The particle characterisation was analysed using one-way analysis of variance (ANOVA) to compare particle size distributions. Fisher’s Least Significant Difference (LSD) and Tukey’s Honestly Significant Difference (HSD) post hoc tests were used to assess significant differences among groups. For dissolution assays, because multiple samples were collected from the same dissolution vessel over time, dissolution data were analysed using repeated-measures ANOVA, with sampling time treated as the within-subject factor and particle type and dissolution medium as between-subject factors. Statistical significance was established at *p* < 0.05. Analyses were conducted using Statistic 14.0.0.15 software (StatSoft Inc., Tulsa, OK, USA).

## 3. Results

### 3.1. Characterisation of Naringenin Particles

**Figure 1:**
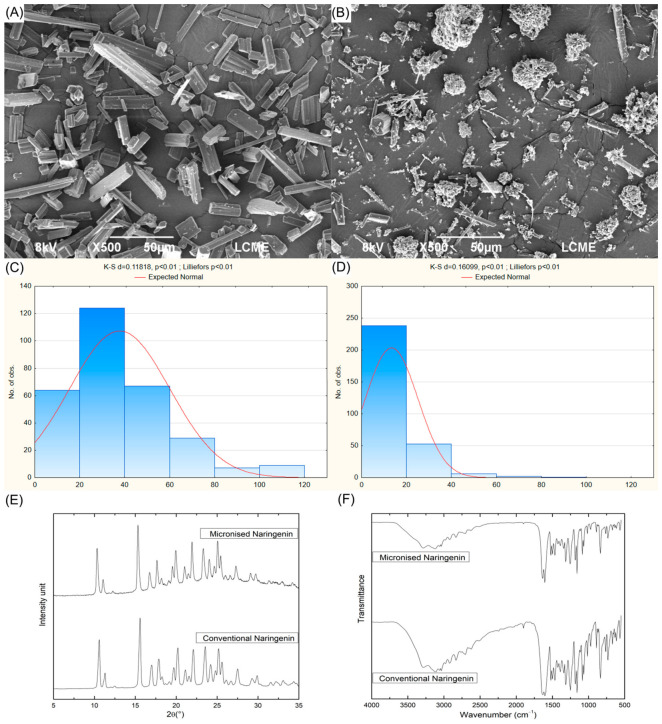
Physicochemical characterisation of conventional and micronised naringenin. SEM micrographs ((**A**) conventional, (**B**) micronised) and particle size distribution analyses ((**C**) conventional, (**D**) micronised) show a reduction in particle size and increased homogeneity after GAS micronisation. XRD and FTIR spectra (**E**,**F**) confirm the preservation of naringenin’s chemical structure.

Conventional naringenin (Figure 1A) exhibited a predominantly prismatic morphology with well-defined edges, typical of crystalline materials. It showed a mean particle size of 37.754 ± 22.315 μm, with a heterogeneous distribution (Figure 1C. For micronised naringenin (Figure 1B), acicular (needle-like) particles were observed, interspersed with others displaying irregular, fragmented shapes. It showed a mean particle size of 13.370 ± 11.770 μm, with a more homogeneous distribution than conventional (Figure 1D; Table 1). Figure 1E presents the diffractograms of conventional and micronised naringenin. The patterns showed diffraction peaks at 11.435°, 12.638°, 15.730°, 17.150°, 18.036°, 18.454°, 19.339°, 19.891°, 20.309°, 21.345°, 21.796°, 22.264°, 23.651°, 24.386°, 24.987°, 25.372°, 25.790°, 26.341°, 26.893°, 27.661°, 29.416°, and 30.051°. When compared with the data reported by Oliveira et al. (2023) [36], who described characteristic peaks for unprocessed naringenin at 10.97°, 11.65°, 15.95°, 18.26°, 20.57°, 22.45°, 25.51°, and 27.86°, a strong agreement between the profiles is observed. Several peaks identified in the present study closely match those reported in the literature.

Both conventional and micronised naringenin exhibited the same characteristic diffraction peaks, indicating that the crystalline structure was preserved after micronisation. The micronised sample showed slightly different peak resolution, possibly due to improved particle dispersion and greater exposure of secondary crystallographic planes. No evidence of amorphisation was observed, confirming the structural stability of naringenin under the applied processing conditions by the GAS technique.

Figure 1F shows the FTIR spectra of conventional and micronised naringenin. The conventional compound exhibited characteristic stretching bands at 3285, 3115, 3060, 1630, 1595, 1520, and 1465 cm^−1^, in agreement with the literature [36,46]. Bands at 3285 and 3115 cm^−1^ correspond to hydroxyl (–OH) groups; 1630 and 1520 cm^−1^ to carbonyl (C=O) stretching; 1460–1630 cm^−1^ to aromatic C=C vibrations; and 1060–1160 cm^−1^ to C–O stretching. No differences were observed between conventional and micronised samples, indicating that the chemical structure of naringenin remained unchanged [36,46,69,70,71].

**Figure 2:**
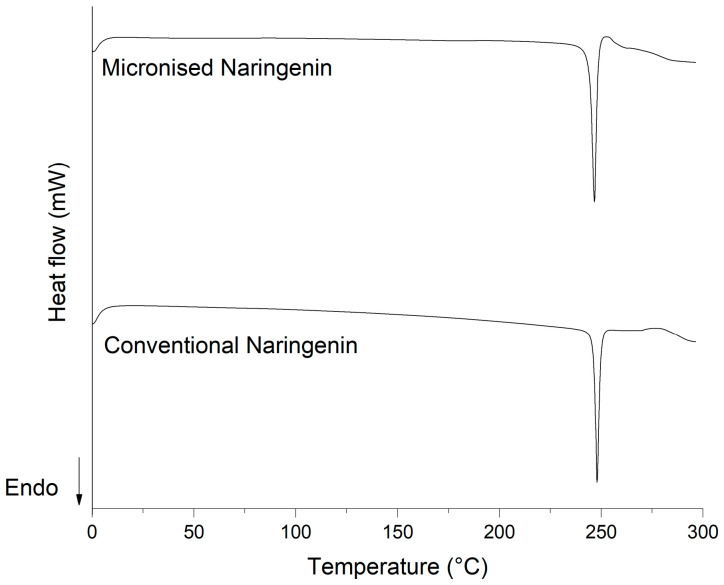
Differential scanning calorimetry (DSC) thermograms of conventional and micronised naringenin. Downward arrows indicate endothermic events (as defined by the convention adopted in this work).

The DSC analyses (Figure 2) of conventional naringenin showed a melting point of 247.94 °C and an enthalpy of fusion (ΔH) of 177.41 J/g. In comparison, micronised naringenin exhibited a melting point of 246.66 °C and a ΔH of 164.18 J/g. These results are consistent with previously reported data [36,46]. The close similarity in melting temperatures, along with comparable enthalpy values, indicates that the micronisation process did not induce any chemical modification of the naringenin structure. This finding is consistent with the results from XRD and FTIR, further supporting the compound’s structural integrity.

### 3.2. Characterisation of Naringin Particles

**Figure 3:**
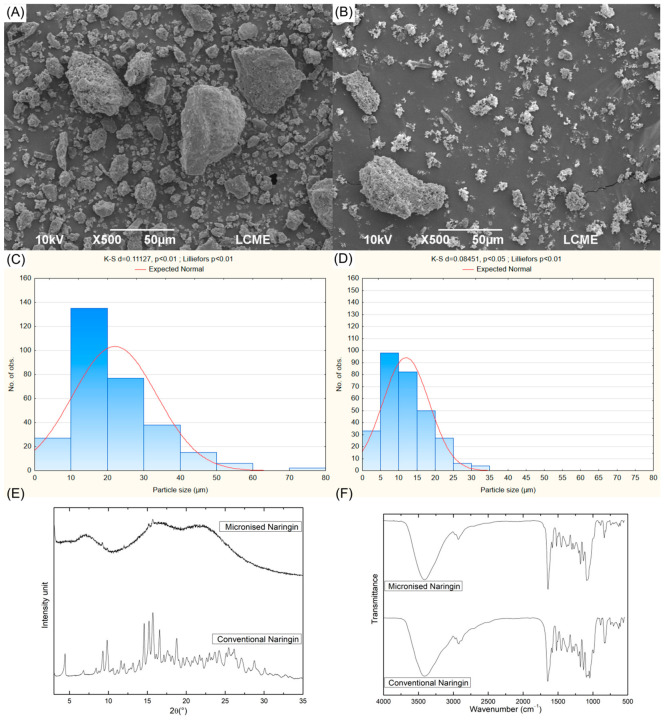
Physicochemical characterisation of conventional and micronised naringin. SEM micrographs ((**A**) conventional, (**B**) micronised) and particle size distribution analyses ((**C**) conventional, (**D**) micronised) show a reduction in particle size and increased homogeneity after GAS micronisation. XRD and FTIR spectra (**E**,**F**) confirm the preservation of naringin’s chemical structure.

SEM analysis of conventional naringin (Figure 3A) revealed particles with irregular morphology, angular and fragmented edges, and no crystalline orientation. Some smaller agglomerates were observed, possibly due to electrostatic cling, but no large clumps formed. The particle size distribution histogram (Figure 3C) showed an average size of 22.040 ± 11.578 μm, with a heterogeneous distribution ranging from particles on the order of 10 μm to larger particles measuring 80 μm. Micronised naringin particles (Figure 3B) appeared smaller and more uniform, with a more rounded, less fractured morphology than conventional naringin. The micronised particles had an average size of 12.007 ± 6.365 μm, showing a more homogeneous size distribution (Figure 3D; Table 2).

The XRD patterns (Figure 3E) of conventional and micronised naringin show characteristic peaks at 4.433°, 9.279°, 9.830°, 14.593°, 15.211°, 15.713°, 16.582°, 18.772°, and 25.473°. These results were compared with previous data for conventional naringin, which has characteristic peaks at 9.87°, 11.64°, 13.22°, 14.62°, 15.72°, 16.63°, 17.60°, 18.76°, and 28.11° [38]. There is good agreement between the crystallographic patterns, particularly for the peaks at 9.8°, 14.6°, 15.7°, 16.6°, and 18.7°, indicating that the analysed sample retains the typical naringin crystal structure. Conventional naringin shows a diffractometric pattern typical of highly crystalline materials, with multiple sharp, well-defined peaks. In contrast, micronised naringin exhibits an amorphous pattern, characterised by the absence of distinct peaks and a broad, diffuse halo [38].

Finally, FTIR analysis (Figure 3F) of conventional naringin identified characteristic bands at 3415, 2925, 1645, 1515, and 1450 cm^−1^. These peaks align with reported values for conventional naringin, such as 3416.65, 2923.60, 1643.33, 1515.63, and 1449.48 cm^−1^, confirming the presence of the main expected functional groups [38,72]. The band at 3415 cm^−1^ corresponds to O-H stretching, typical of hydroxyl groups in both the glycosidic part and the flavonoid aglycone. The peak at 2925 cm^−1^ refers to the stretching of aliphatic C-H bonds. The band at 1645 cm^−1^ corresponds to C=O stretching, while the peaks at 1515 and 1450 cm^−1^ are associated with vibrations of aromatic rings and deformations of the molecule’s carbon skeleton. In comparison, the spectrum of the micronised sample showed a decrease in the intensity of bands near 1150 cm^−1^, attributed to C–O–C glycosidic bonds.

**Figure 4:**
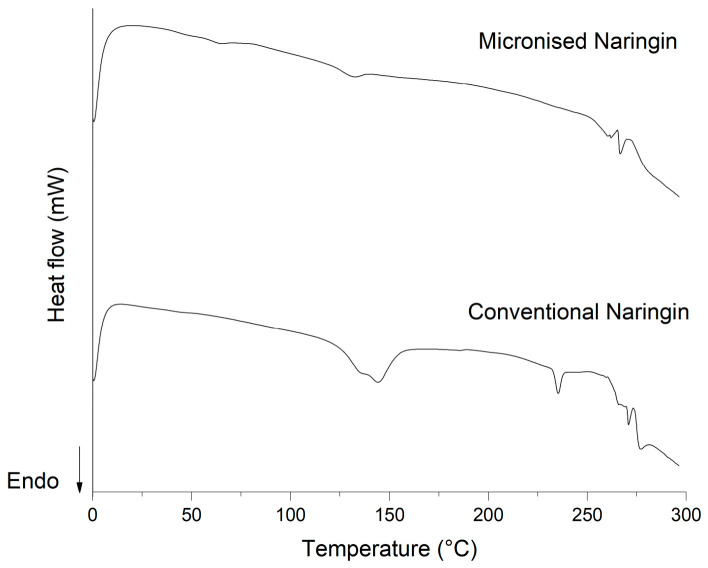
Differential scanning calorimetry (DSC) thermograms of conventional and micronised naringin. Downward arrows indicate endothermic events (as defined by the convention adopted in this work).

The DSC thermograms (Figure 4) clearly distinguish between conventional and micronised naringin. The conventional sample exhibited a melting point of 144.18 °C and an enthalpy of fusion (ΔH) of 62.02 J/g, indicating a well-defined crystalline structure. In contrast, the micronised naringin showed a lower melting temperature (131.69 °C) and a markedly reduced ΔH (5.94 J/g), suggesting a significant decrease in crystallinity after GAS processing. This reduction in enthalpy, together with the slight shift in melting point, indicates disruption of the ordered lattice and the formation of less crystalline or partially amorphous structures. These findings are consistent with XRD results showing decreased peak intensity and with SEM observations of reduced particle size, confirming that micronisation effectively alters the physical structure without compromising the chemical integrity of naringin. Similar behaviour has been reported by Oliveira et al. (2024) [38] for naringin processed under supercritical conditions, reinforcing the impact of the GAS technique on reducing crystallinity and modifying thermal properties.

### 3.3. Dissolution Profile of Conventional or Micronised Naringin and Naringenin

**Figure 5:**
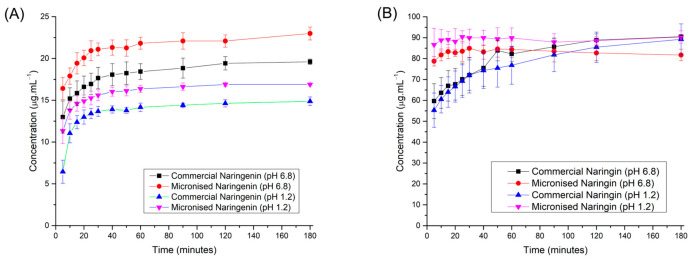
Apparent dissolution profiles of conventional and micronised naringenin (**A**) and naringin (**B**) in acidic (pH 1.2) and phosphate buffer (pH 6.8) media at 37 ± 0.5 °C. Error bars represent the standard deviation of three replicates (n = 3).

The apparent dissolution profiles of naringenin and naringin (Figure 5) were analysed separately using repeated-measures ANOVA, considering sampling time as the within-subject factor and dissolution medium and particle type as between-subject factors. For naringenin, significant effects were observed for time (*p* < 0.000001), dissolution medium (*p* = 0.000011), and particle type (*p* = 0.000455). Significant interactions between time and medium (*p* = 0.000310), time and particle type (*p* = 0.003083), and among time, medium, and particle type (*p* = 0.000470) were also detected, indicating that the effect of micronisation depended on both dissolution medium and sampling time. For naringin, significant effects of time (*p* < 0.000001) and particle type (*p* = 0.004763) were observed, whereas dissolution medium had no significant effect (*p* = 0.602887). A significant interaction between time and particle type (*p* < 0.000001) was detected, demonstrating that micronisation altered the dissolution profile throughout the experiment regardless of the dissolution medium. Overall, micronised naringenin and naringin exhibited faster initial apparent dissolution and higher apparent dissolved concentrations than their respective conventional counterparts, particularly during the early stages of the dissolution assay. For naringenin, apparent dissolution was notably higher in the phosphate buffer (pH 6.8) than in the acidic medium (pH 1.2), reflecting its weakly acidic nature and increased ionisation at higher pH. The micronised naringenin sample reached approximately 22 μg·mL^−1^ after 180 min, whereas the conventional form plateaued at approximately 18 μg·mL^−1^. Conversely, under acidic conditions, both samples showed slower apparent dissolution and lower equilibrium concentrations (15 and 12 μg·mL^−1^ for processed and conventional forms, respectively) (Figure 5A). For naringin, the apparent dissolution profiles indicate a high dissolution profile in both media, with slightly faster dissolution in the acidic medium (pH 1.2). The micronised samples exhibited a steeper initial apparent dissolution rate, reaching equilibrium (90 μg·mL^−1^) sooner than the conventional form due to reduced particle size and increased surface area from micronisation. At pH 6.8, both samples showed slower dissolution compared to pH 1.2, likely due to decreased naringin ionisation at near-neutral pH (Figure 5B).

### 3.4. Naringin and Naringenin Can Interact with TRPV1 Channels

**Figure 6:**
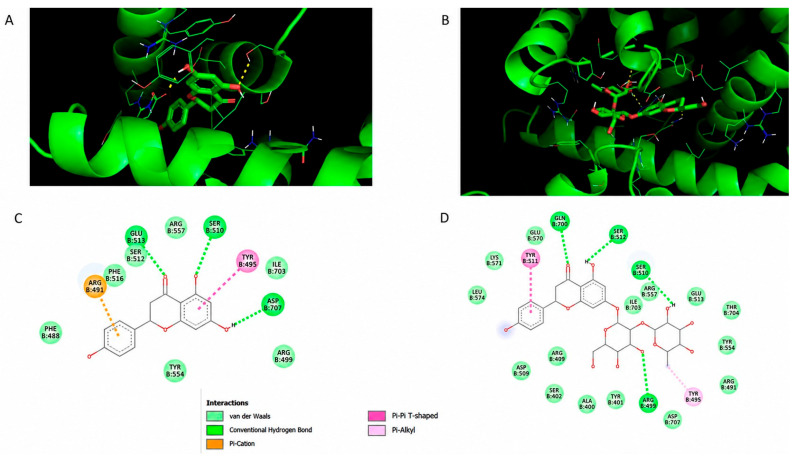
Molecular docking for TRPV1-naringenin (**A**,**C**) and TRPV1-naringin (**B**,**D**).

Molecular docking showed that naringenin and naringin can fit into the TRPV1 channel cavity in their best docking poses (Figure 6). The naringenin-TRPV1 complex was stabilised through hydrogen bonds with GLU: B 513, SER B:510, and ASP B:707, indicating strong polar interactions. A notable pi-cation interaction was identified between the aromatic ring of naringenin and ARG B:491, suggesting electrostatic stabilisation. The ligand also exhibited pi-pi T-shaped stacking with TYR B: 495, contributing to hydrophobic stabilisation. Van der Waals interactions with residues including PHE B:488, PHE B:516, SER B:512 TYR B 554, ARG B:499, ARG B:557, ILE B:703, and others provided additional stabilisation by promoting close contacts (Figure 6A,C).

Molecular docking of TRPV1 with naringin revealed a complex network of stabilising interactions within the receptor’s binding site. Naringin formed multiple conventional hydrogen bonds with key residues such as GLN B:700, SER B:512, SER B:510, and ARG B:499, indicating strong polar interactions that help anchor the molecule. Hydrophobic interactions included a pi-pi T-shaped interaction with TYR B: 511 and a pi-alkyl interaction with TYR B: 495, supporting binding through nonpolar contacts. Several van der Waals interactions were identified with surrounding residues, including LYS B: 571, GLU B:570, LEU B:574, ASP B:509, ARG B:409, SER B:402, ALA B:400, TYR B:401, ASP B:707, ARG B:491, TYR B: 554, THR B:704, GLU B:513, and ILE B:703, reinforcing close-range molecular complementarity (Figure 6B,D).

The free energy of binding (affinity) for the docking of TRPV1 with the naringenin complex was F.E.B = −8.8 kcal/mol, and the estimated inhibition constant was Ki = 354.46 nM, while for the TRPV1 complex with naringin, the F.E.B was −10.0 kcal/mol, and Ki was 46.77 nM. The R.M.S.D. was less than 2 Å for all systems studied. Docking scores and estimated Ki values should be interpreted only as computational, hypothesis-generating indicators of possible ligand–TRPV1 interactions and do not represent experimentally determined binding affinities, thermodynamic parameters, or inhibitory potency.

### 3.5. Micronised Naringin and Naringenin Reduce Capsaicin-Induced Nociception Without Causing Adverse Effects

**Figure 7:**
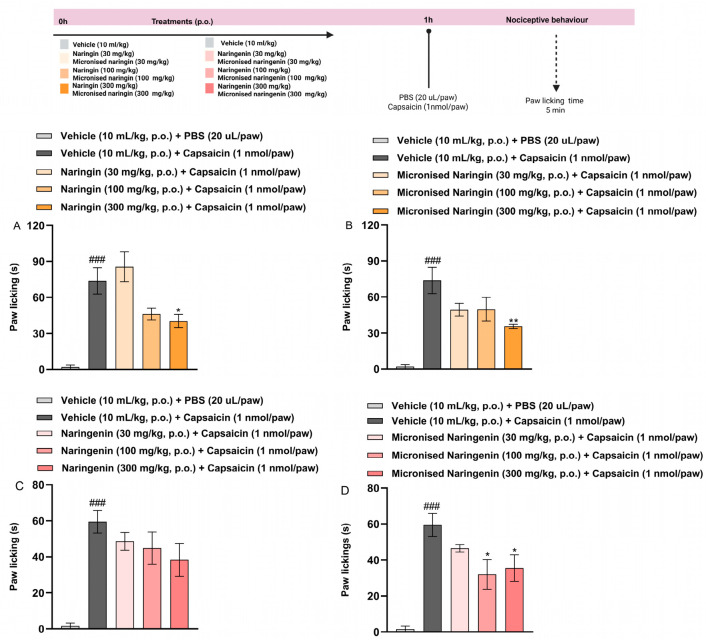
Effects of conventional and micronised naringin and naringenin on capsaicin-induced nociception. Paw licking time (seconds; s) caused by intraplantar injection of PBS (20 μL/paw, i.pl.) or capsaicin (1 nmol/paw, i.pl.) in animals orally treated with vehicle (10 mL/kg, p.o.), conventional (**A**) or micronised (**B**) naringin (30, 100, 300 mg/kg, p.o.), or conventional (**C**) or micronised (**D**) naringenin (30, 100, 300 mg/kg, p.o.). Data are shown as mean ± SEM (n = 6 animals). The vehicle + PBS group data represent the same animals across all graphs. ^###^ *p* < 0.001 compared to the oral vehicle + PBS group. * *p* < 0.05; ** *p* < 0.01 compared to the vehicle + capsaicin group; one-way ANOVA followed by Bonferroni post hoc test. Experimental schema created in BioRender. Zilli, G. A. (2026) https://BioRender.com/7okwc2a.

Intraplantar administration of capsaicin increased paw licking time compared to animals that received PBS intraplantar, indicating the development of nociception (Figure 7). Conventional naringin at a dose of 300 mg/kg, but not at 100 or 30 mg/kg, reduced capsaicin-induced nociception compared to vehicle-treated animals that received intraplantar capsaicin, with an inhibition of 45.4 ± 22% (Figure 7A). Micronised naringin also decreased capsaicin-induced nociception only at 300 mg/kg, with an inhibition of 52 ± 4% (Figure 7B). Conversely, conventional naringenin was unable to prevent capsaicin-induced nociception (Figure 7C), while micronised naringenin (100 and 300 mg/kg) reduced nociception with inhibitions of 46.2 ± 14% and 40.3 ± 24%, respectively (Figure 7D).

Furthermore, three doses (30, 100, 300 mg/kg, p.o.) of both conventional and micronised naringin and naringenin did not cause adverse effects on locomotor activity or sedation, nor did they alter body temperature in mice compared with the vehicle group (Table 3). We selected an intermediate dose of 100 mg/kg for the following experiments, also based on previous studies with naringin and micronised naringenin, which ranged from 50 to 200 mg/kg [36,38].

### 3.6. Effect of Conventional or Micronised Naringin and Naringenin on the TRPV1 mRNA Expression in the Paclitaxel-Induced Pain Model

**Figure 8:**
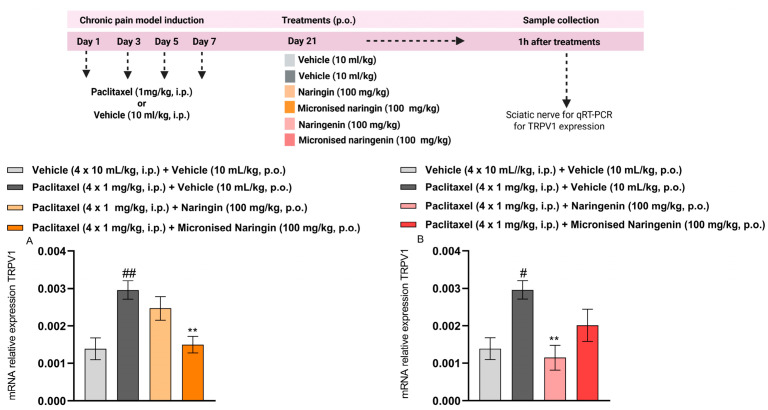
Effect of micronised naringin and naringenin on TRPV1 mRNA expression in the paclitaxel-induced chronic pain model. Sciatic nerve samples were collected from animals that received vehicle (10 mL/kg, p.o.), conventional naringin (**A**) or naringenin (**B**) (100 mg/kg, p.o.), or micronised naringin (**A**) or naringenin (**B**) (100 mg/kg, p.o.) at 21 days after the first repeated paclitaxel administration (1 + 1 + 1 + 1 mg/kg, i.p.) or vehicle (4 × 10 mL/kg, i.p.). Data are presented as mean ± SEM (n = 6 animals; n = 5 pacli + vehi group due to an outlier defined by Grubbs’ statistical test). The data for the vehicle + vehicle group and the paclitaxel + vehicle group represent the same animals in both graphs. ^#^ *p* < 0.05; ^##^ *p* < 0.01 compared to the vehicle (i.p.) group; ** *p* < 0.01 compared to the vehicle (p.o.) group; one-way ANOVA followed by Bonferroni’s post hoc test. Experimental schema created in BioRender. Zilli, G. A. (2026) https://BioRender.com/u88ye56.

Repeated administration of paclitaxel increased TRPV1 mRNA expression in the sciatic nerve of mice. Micronised naringin, but not conventional naringin, was able to decrease this increase with 93 ± 13% inhibition (Figure 8A). Naringenin reduced the increase in TRPV1 mRNA expression by 100%, while micronised naringenin, although not significantly, showed a 60 ± 18% reduction (Figure 8B).

### 3.7. Conventional or Micronised Naringin and Naringenin Alleviate Paclitaxel-Induced Mechanical and Cold Sensitivity in the Chronic Pain Model

**Figure 9:**
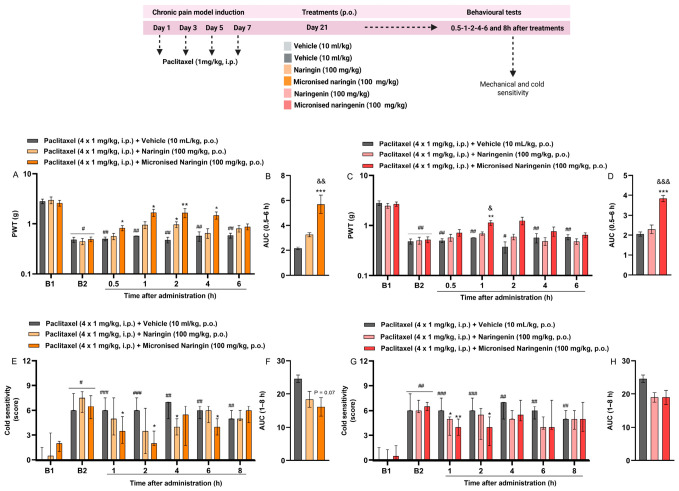
Micronised naringin and naringenin reduce mechanical (**A**–**D**) and cold sensitivity (**E**–**H**) in the paclitaxel-induced chronic pain model. Time response curves show animals that received vehicle (10 mL/kg, p.o.), conventional naringin or naringenin (100 mg/kg, p.o.), or micronised naringin or naringenin (100 mg/kg, p.o.) at 21 days after the first paclitaxel dose (1 + 1 + 1 + 1 mg/kg, i.p.). B1 represents the basal mechanical threshold or cold sensitivity before paclitaxel, while B2 indicates the baseline mechanical threshold or cold sensitivity after paclitaxel and before treatments. Data are expressed as mean ± SEM (**A**–**D**; **F**,**H**) or median with interquartile range (**E**,**G**) (n = 6 animals; n = 5 pacli + vehi group due to limited animal availability from the breeding facility). The data for the paclitaxel + vehicle group represent the same animals in the graphs. ^#^ *p* < 0.05; ^##^ *p* < 0.01; ^###^ *p* < 0.001 compared to B1; * *p* < 0.05; ** *p* < 0.01; *** *p* < 0.001 compared to the vehicle group; ^&^ *p* < 0.05; ^&&^ *p* < 0.01; ^&&&^ *p* < 0.001 compared to the paclitaxel + conventional naringin or naringenin group; one-way or two-way repeated measures ANOVA (**A**–**D**; **F**,**H**); or mixed-effects model (REML) (**E**,**G**) followed by Bonferroni’s post hoc test. Experimental schema created in BioRender. Zilli, G. A. (2026) https://BioRender.com/9rcdxq4.

Repeated (chronic) paclitaxel administration decreased the mechanical PWT in response to von Frey filaments and increased cold sensitivity compared to baseline (B1) at 21 days after the first administration, indicating the development of mechanical and cold allodynia. Conventional naringin reduced paclitaxel-induced mechanical allodynia only 2 h after administration, with a 20.9 ± 5% inhibition compared to the vehicle group. In contrast, micronised naringin showed effects from 0.5 to 4 h after administration, with 50.9 ± 11% inhibition at 2 h (Figure 9A). Conventional naringenin did not reduce paclitaxel-induced mechanical allodynia. However, micronised naringenin alleviated mechanical allodynia only at 1 h, compared with the vehicle and conventional naringenin groups, with an inhibition of 25.1 ± 4% (Figure 9B).

Conventional naringin decreased paclitaxel-induced cold allodynia only at 4 h after administration, with a 39.2 ± 6% inhibition compared to the vehicle group. Micronised naringin showed effects at 1, 2, and 6 h after administration, with an inhibition of 62.7 ± 17% at 2 h (Figure 9E). Similarly, conventional naringenin reduced paclitaxel-induced cold allodynia only at 1 h after its administration, with a 35.8 ± 8% inhibition, while micronised naringenin was effective from 1 to 2 h, with an inhibition of 48.8 ± 13% at 2 h (Figure 9G).

### 3.8. Conventional or Micronised Naringin and Naringenin Reduce Paclitaxel-Induced Mechanical and Cold Sensitivity in the Acute Pain Model

**Figure 10:**
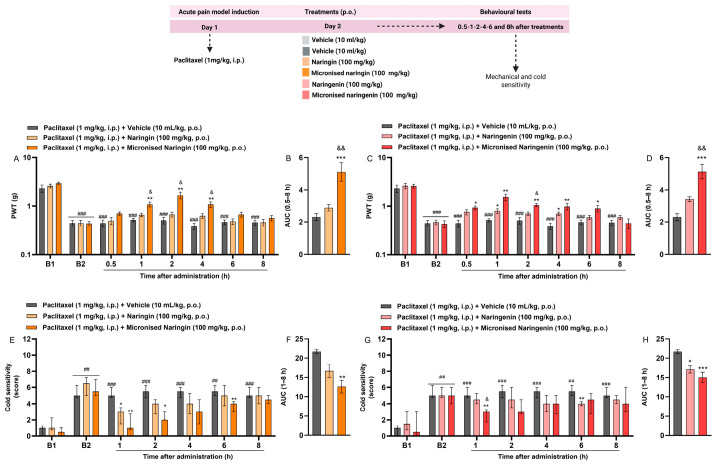
Micronised naringin and naringenin reduce mechanical (**A**–**D**) and cold sensitivity (**E**–**H**) in the paclitaxel-induced acute pain model. Time response curves in animals that received vehicle (10 mL/kg, p.o.), conventional naringin or naringenin (100 mg/kg, p.o.), or micronised naringin or naringenin (100 mg/kg, p.o.) at 1 day after acute paclitaxel administration (1 mg/kg, i.p.). B1 indicates basal mechanical threshold or cold sensitivity before paclitaxel administration, while B2 shows the basal mechanical threshold or cold sensitivity after paclitaxel administration and before treatments. Data are expressed as mean ± SEM (**A**–**D**; **F**,**H**) or median with interquartile range (**E**,**G**) (n = 6 animals). The data for the paclitaxel + vehicle group represent the same animals in the graphs. ^##^ *p* < 0.01; ^###^ *p* < 0.001 compared to B1; * *p* < 0.05; ** *p* < 0.01; *** *p* < 0.001 compared to the vehicle group; ^&^ *p* < 0.05 ^&&^ *p* < 0.01 compared to the paclitaxel + conventional naringin or naringenin group; one-way or two-way repeated measures ANOVA (**A**–**D**; **F**,**H**); or mixed-effects model (REML) (**E**,**G**) followed by Bonferroni’s post hoc test. Experimental schema created in BioRender. Zilli, G. A. (2026) https://BioRender.com/e313llt.

A single (acute) dose of paclitaxel decreased the mechanical PWT and increased cold sensitivity compared to baseline (B1) at 24 h post-administration, indicating mechanical and cold allodynia in the acute pain model. Conventional naringin was not able to reduce paclitaxel-induced mechanical allodynia. In contrast, micronised naringin showed effects from 1 to 4 h after administration compared to vehicle or conventional naringin, with an inhibition of 60.2 ± 12% at 2 h (Figure 10A). Conventional naringenin decreased paclitaxel-induced mechanical allodynia at 1 and 4 h after administration, with an inhibition of 15.1 ± 3% at 1 h. Micronised naringenin exhibited effects from 0.5 to 6 h, reaching 53 ± 10% inhibition at 1 h (Figure 10B). Notably, micronised naringenin showed a more substantial antinociceptive effect than conventional naringenin at 2 h (Figure 10B).

For cold allodynia, conventional naringin was only effective 1 h after administration, with a 54.7 ± 15% inhibition compared to the vehicle group. Micronised naringin, however, reduced cold allodynia at 1, 2, and 6 h after administration, with an inhibition of 76.6 ± 16% at 1 h (Figure 10E). Conventional naringenin was unable to reduce paclitaxel-induced cold allodynia, whereas micronised naringenin was effective at 1 and 6 h after administration, compared with the vehicle and conventional naringin (at 1 h), with an inhibition of 54.7 ± 9% at 1 h (Figure 10G).

### 3.9. Micronised Naringin and Naringenin Decrease Paclitaxel-Induced Heat Hyperalgesia

**Figure 11:**
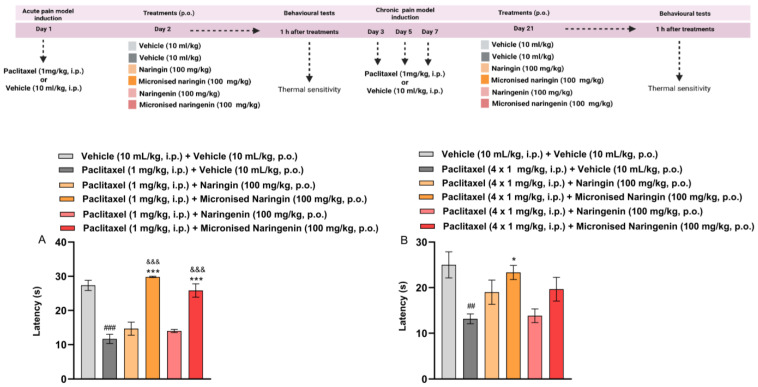
Micronised naringenin and naringin reduce paclitaxel-induced thermal hyperalgesia. Paw response latency was measured after administering either vehicle (10 mL/kg, p.o.), conventional naringin or naringenin (100 mg/kg, p.o.), or micronised naringin or naringenin (100 mg/kg, p.o.) in the acute model (**A**) or chronic/neuropathic model (**B**) of paclitaxel-induced pain. Data are expressed as mean ± SEM (n = 6 animals; n = 5 pacli + micro naringin and pacli + micro naringenin groups due to an outlier defined by Grubbs’ statistical test). The same animals from the acute model were reused following the same induction protocol as the chronic model. ^##^ *p* < 0.01; ^###^ *p* < 0.001 compared to the vehicle + vehicle group; * *p* < 0.05; *** *p* < 0.001 compared to the paclitaxel + vehicle group; ^&&&^ *p* < 0.001 compared to the paclitaxel + conventional naringin or naringenin group. One-way ANOVA followed by Bonferroni’s post hoc test. Experimental schema created in BioRender. Zilli, G. A. (2026) https://BioRender.com/42n991h.

Acute or repeated administration of paclitaxel reduced paw withdrawal latency in the hot plate test compared with the vehicle group, confirming heat hyperalgesia (Figure 11). Only micronised forms of naringin or naringenin were able to reverse paclitaxel-induced heat hyperalgesia during the acute phase, compared with groups treated orally with the vehicle or conventional forms, with inhibition rates of 100% and 90.5 ± 12%, respectively (Figure 11A). In the chronic phase, only micronised naringin alleviated heat hyperalgesia compared to the oral vehicle group, with an inhibition of 86 ± 13% (Figure 11B).

## 4. Discussion

Global cancer cases are expected to increase [1], likely leading to higher chemotherapy use and adverse effects caused by them, such as paclitaxel-induced pain, which significantly impacts quality of life and has limited treatment options [4,5,6,7,8,15]. Consequently, new therapeutic strategies are needed to alleviate these adverse effects. Naringin and its aglycone, naringenin, display multiple activities, including antioxidant, anti-inflammatory, anticancer, and antinociceptive effects [23,24,25,26,27,29,30,40], making them promising candidates for research in cancer pain management. However, their low oral bioavailability remains a major obstacle, limiting their therapeutic potential [24].

Our findings revealed that micronised naringin and naringenin, produced through supercritical fluid processing, improved their pharmacological profiles in a paclitaxel-induced pain model. The micronised forms had higher in vitro apparent dissolution rates than the conventional forms. Moreover, we confirm that naringin binds to TRPV1 channels via molecular docking [34] and also demonstrate that naringenin interacts with this channel. The molecules reduced capsaicin (a TRPV1 agonist)-induced nociception and decreased TRPV1 expression in the sciatic nerve, strengthening the idea that TRPV1 is a probable contributor to these effects. Since TRPV1 is involved in pathological pain, including chemotherapy-induced pain [7,35], we showed that orally administered micronised naringin and naringenin exhibited better antinociceptive effects than conventional forms in the paclitaxel-induced pain model. Although further research is necessary, micronised naringin and naringenin appear promising for alleviating paclitaxel-induced pain, with TRPV1 likely contributing to the observed antinociceptive effects. These findings emphasise the importance of pharmaceutical techniques such as micronisation in unlocking the therapeutic potential of flavonoids for pain management.

Micronisation of naringin and naringenin using the GAS technique decreased the mean particle size while preserving the chemical structure of the molecules, corroborating previous data [36,38]. Although the conventional form of naringin had an apparent dissolution rate similar to the micronised form after one hour, the micronised form showed this characteristic within the first few minutes. Already, micronised naringenin demonstrated a better apparent dissolution rate throughout the entire testing period. These findings confirm the effectiveness of the micronisation process, mainly due to smaller particle size and increased surface area, which improve solid–liquid contact and mass transfer efficiency [36,38,42,73]. Consistent with this, micronisation of naringin using other supercritical antisolvent techniques has been shown to improve permeation profiles and increase plasma and tissue concentrations compared with the conventional form [74,75]. Additionally, a naringenin–betaine cocrystal produced via the GAS technique demonstrated increased permeability across the blood–brain and intestinal barriers in vitro at low concentrations, thereby supporting efficacy and safety [76]. Collectively, these findings reinforce the potential of supercritical processing to enhance the biopharmaceutical performance of poorly soluble compounds, in agreement with our results. However, further pharmacokinetic studies are still needed.

Eom and colleagues (2021) [34] found that naringin binds to TRPV1 at amino acid residues D471 and N628 and selectively inhibits capsaicin-stimulated inward currents in *Xenopus oocytes*, suggesting that naringin acts as an antagonist, which could help develop analgesics targeting TRPV1. At high concentrations, naringenin also partially blocked TRPV1-mediated calcium influx in HEK293 cells expressing this channel [77]. TRPV1 channels are present in nociceptive pathways, functioning as molecular detectors that activate sensory neurons to produce acute or persistent pain [35], including that caused by paclitaxel [7,17,18,19,20]. Notably, recent studies have demonstrated that naringenin and a naringenin-derived flavanone exhibit antinociceptive effects in a paclitaxel-induced pain model [28,29]. Therefore, we examined the role of TRPV1 in the antinociceptive effects of naringin and naringenin on paclitaxel-induced pain. Our data from molecular docking studies indicated that both naringin and naringenin bind to the TRPV1 channel, extending the findings reported by Eom and colleagues (2021) [34] for naringin. However, further research is needed to determine whether these molecules act as TRPV1 antagonists.

Our antinociceptive screening tests were also consistent with an interaction between naringin and naringenin and TRPV1. Here, capsaicin, a TRPV1 agonist, induced nociceptive paw-licking behaviour, as previously reported in other studies [39,59,78,79]. Conventional and micronised naringin both decreased capsaicin-induced nociception. However, only the micronised form of naringenin was effective against capsaicin-induced nociception, suggesting that micronisation may enhance this effect. Our findings align with Pinho-Ribeiro et al. (2016) [25], who showed that naringenin reduced overt pain-like behaviours induced by acetic acid, phenyl-p-benzoquinone, formalin, and capsaicin. The presence of naringin in a plant extract was also linked to antinociceptive effects in formalin- and acetic acid-induced acute models [80].

Additionally, we observed paclitaxel-induced upregulation of TRPV1 mRNA in the sciatic nerve of mice, consistent with reports of increased TRPV1 in DRG neurons and the spinal cord in this model [18,19]. The upregulation of TRPV1 has also been described in models induced by oxaliplatin [81], cisplatin [82], gout [83], and sciatic nerve injury [84]. Micronised naringin and conventional naringenin decreased paclitaxel-induced TRPV1 upregulation, and although not statistically significant, micronised naringenin showed a tendency toward an effect. Analysing protein expression via Western blotting, immunohistochemistry, or receptor-binding studies could better clarify the impact on the channels, especially if micronised molecules behave similarly to conventional ones or if micronisation alters these properties. These findings are consistent with a previous study showing that naringenin reduced TRPV1 upregulation in DRG neurons of mice in a bone cancer pain model [26]. Naringenin also decreased TRPV1 upregulation in the constipation model [85]. Moreover, naringin alleviated UVB-induced senescence and damage in keratinocytes by inhibiting TRPV1 and reducing its upregulation [86].

These findings suggest that TRPV1 probably contributes to the antinociceptive effects of naringin and naringenin. Although further studies are needed to confirm this hypothesis, naringenin and a naringenin-derived trimethoxyflavanone exhibited antinociceptive effects in a paclitaxel-induced pain model involving CGRP signalling [28,29], and a link between TRPV1 and CGRP has already been demonstrated in this model [32,33]. Cultures of DRG neurons pretreated with low concentrations of paclitaxel and stimulated with capsaicin showed increased CGRP release, whereas high concentrations reduced transmitter release, an effect associated with desensitisation [32]. Moreover, Wang and colleagues (2022) [33] demonstrated that paclitaxel upregulated TRPV1 in the DRG, primarily in small-sized CGRP- and IB-4-positive sensory neurons. TRPV1 is also involved in CGRP release in oxaliplatin-induced pain and migraine models [30,31]. Notably, reducing TRPV1 expression, silencing, or antagonising (with capsazepine and SB-366791) has helped alleviate paclitaxel-induced nociceptive behaviours, such as mechanical and heat sensitivity [18,19,87,88]. Therefore, we assess whether oral administration of micronised naringin and naringenin could also produce this effect in paclitaxel-induced pain syndrome.

The antinociceptive effect of naringenin on paclitaxel-induced pain has been examined previously [28,29]. However, Mei and colleagues (2023) [28] found that a naringenin-derived flavanone, trimethoxyflavonone, showed stronger antinociceptive effects than naringenin when administered intrathecally. The study by Pan et al. (2024) [29] also observed antinociceptive effects in this model, but naringenin was administered intraperitoneally. These studies may have employed methods other than oral administration due to issues with bioavailability. The review by Memariani and colleagues (2021) [24] explains that naringenin’s low solubility limits its absorption in the gastrointestinal tract after oral administration, resulting in a bioavailability of approximately 15%. It also states that the oral bioavailability of naringin in humans is low, estimated at around 9% [24]. Oral administration is the most commonly used route and is preferred by patients due to its simplicity, non-invasiveness, and convenience. However, its effectiveness is often limited by poor drug solubility and permeability across mucosal barriers, emphasising the need for new pharmaceutical strategies to address these issues [89]. Therefore, we examined the oral effects of the micronised form of naringenin and of its glycosylated form, naringin, to determine whether the glycosylated form might be more effective, since naringin is converted to naringenin in the gastrointestinal tract [24].

Existing studies also assess only mechanical and heat sensitivity within 1 to 7 days after a single paclitaxel administration [28,29]. However, chemotherapy-induced pain is characterised by mechanical and heat sensitivity, as well as cold allodynia [3,5,7,8,9,11]. Moreover, clinically, this condition follows a biphasic course: an acute pain phase that develops within the first hours to days after paclitaxel infusion, followed by a chronic neuropathic phase that may persist for weeks, months, or even years after cessation of chemotherapy [5,7,8,10,12,13]. Therefore, models have attempted to replicate both the acute pain occurring in the first hours after a single dose of paclitaxel and the neuropathic pain observed following repeated doses [16,58,90,91,92]. Consistent with these studies, paclitaxel induced mechanical and cold allodynia, as well as heat hyperalgesia, at both the acute and chronic stages. In both phases, micronised forms of naringin and naringenin demonstrated superior effects compared to conventional forms, which, in some cases, failed to show efficacy. Notably, TRPV1 is activated by noxious heat, contributing to thermal hypersensitivity under pathological conditions [35], reinforcing the hypothesis that this channel probably contributes to the antinociceptive effects of naringin and naringenin. These data support previous research showing enhanced dissolution and pharmacological effects of micronised forms of naringin and naringenin in models predictive of positive symptoms of schizophrenia [36,38]. The micronisation of compounds using supercritical CO_2_ has been applied to other molecules, such as curcumin, resveratrol, and luteolin, which have demonstrated potential benefits in zebrafish seizure models compared to conventional forms [93,94,95].

Our findings also support other studies, demonstrating the antinociceptive potential of naringenin and naringin, which are mainly used for repeated treatments and administered intraperitoneally or intrathecally. Naringin decreased mechanical and thermal sensitivity through anti-inflammatory and antioxidant mechanisms in the streptozotocin-induced diabetic neuropathic pain model [40], and it also showed anti-inflammatory effects in a monosodium iodoacetate-induced osteoarthritis model [27]. Additionally, naringin demonstrated neuroprotective effects after spinal cord injury in rats, improving mechanical, cold and heat hypersensitivity and reducing inflammatory, oxidative, and histopathological damage [96]. Naringenin reduced mechanical hyperalgesia in inflammatory pain induced by carrageenan, capsaicin, and CFA by decreasing oxidative stress, neutrophil recruitment, and hyperalgesic cytokines, as well as suppressing NFκB activation and activating the NO-cyclic GMP-PKG-ATP-sensitive K+ channel pathway [25]. Naringenin alleviated heat and mechanical sensitivity and improved bone damage in a bone cancer pain model through the NF-κB/uPA/PAR2 pathway and by reducing TNF-α [26]. Additionally, naringenin reduced mechanical allodynia and oxidative stress, while promoting spinal microglia M2 polarisation in a cancer-induced bone pain model by regulating the AMPK/PGC-1α signalling axis [23]. Moreover, naringenin reduced thermal and mechanical sensitivity in diabetic neuropathic pain through antioxidant and anti-inflammatory effects [97]. Naringenin also decreased mechanical hyperalgesia and oedema in the zymosan-induced arthritis model by inhibiting leucocyte infiltration, oxidative stress, inflammatory compounds, and NFκB activation [98]. Taken together, these results demonstrate that most of the antinociceptive effects of naringin and naringenin are mediated through their antioxidant and anti-inflammatory properties. These effects may also explain relief from paclitaxel-induced pain, as this pain involves inflammatory and oxidative components, TRPV1 channels, and other mechanisms [7].

Pharmacological options for chemotherapy-induced pain provide limited benefits [5]. Importantly, none of the tested regimens, whether conventional or micronised, caused sedation, locomotor impairment, or changes in body temperature. Maintaining locomotor activity is essential to ensure that antinociceptive effects are not mistaken for locomotor impairment or sedation, which could prevent paw withdrawal. Furthermore, our data and those of Eom et al. (2021) [34] suggest that naringin and naringenin may act as TRPV1 antagonists, underscoring the importance of maintaining body temperature. A key limitation of TRPV1 as a therapeutic target is its role in thermoregulation. Systemic antagonists often induce hyperthermia by blocking the proton-activation mode, whereas heat-mode blockade has minimal impact [35,99,100]. Our finding that rectal temperature remains unchanged is therefore a strength, suggesting that these flavonoids do not fully inhibit the proton mode (unlike failed clinical candidates such as AMG517) [99] and indicating a potentially safer, modality-selective profile. The review by Memariani and colleagues (2021) [24] also compiles multiple studies indicating that naringin and naringenin are relatively safe or non-toxic, even being tested in some clinical trials both alone and in the natural composition of orange juice. However, further research is required to understand the long-term effects of these molecules in humans.

Paclitaxel-induced pain syndrome is a debilitating condition [4,5,6,7,8] that requires better management. We demonstrated that micronised naringin and naringenin, prepared using supercritical technology, reduced the mean particle size while preserving the chemical characteristics of the molecules, and exhibited improved in vitro apparent dissolution profile compared with their conventional forms. The micronised molecules alleviated nociceptive behaviours in both the acute and chronic phases of paclitaxel-induced pain syndrome when administered orally, a route of significant clinical relevance [89]. One limitation of our study is that it involved only male mice. However, despite dimorphisms in mechanisms, particularly neuroimmune, paclitaxel induces mechanical and thermal sensitivity similarly in both males and females [29,101], and most studies use males to establish this model [87,88,102]. Furthermore, the antinociceptive effect of intraperitoneal naringenin did not differ between the sexes in the paclitaxel-induced neuropathic pain model [29]. However, future studies should include both sexes to explore mechanistic or pharmacokinetic approaches. Although the absence of pharmacokinetic assays also limits our study, the findings suggest that micronisation improved apparent dissolution and was associated with increased oral antinociceptive efficacy. While more specific assays are needed, docking studies, antinociceptive effects in the capsaicin test, and reductions in channel expression suggest that TRPV1 channels are a probable contributor to the observed antinociceptive effects.

Additionally, it is important to consider how analgesics impact tumour progression [103]. In this context, naringin and naringenin could be advantageous, as they exhibit antitumour effects alone or in combination with chemotherapeutic agents such as paclitaxel [24]. Therefore, micronised naringin and naringenin appear promising for alleviating paclitaxel-induced pain syndrome when taken orally. Naringenin is especially interesting because its effects in this model have already been studied, and it is a smaller molecule than naringin, which could make further development and optimisation easier.

## Figures and Tables

**Table 1:** Mean particle size and morphology of the naringenin particles.

Particle	Mean Particle Size (μm)	Morphology	Solid-State
Conventional Naringenin	37.754 ± 22.315 ^a^	Regular prismatic	Crystalline
Micronised Naringenin	13.370 ± 11.770 ^b^	Fragmented, prismatic, and needle-like	Crystalline

Different letters indicate statistically significant differences according to Tukey and Fisher tests.

**Table 2:** Mean particle size and morphology of the naringin particles.

Particle	Mean Particle Size (μm)	Morphology	Solid-State
Conventional Naringin	22.040 ± 11.578 ^a^	Irregular prismatic	Crystalline
Micronised Naringin	12.007 ± 6.365 ^b^	Agglomerates of small spherical particles	Partially amorphous

Different letters indicate statistically significant differences according to Tukey and Fisher tests.

**Table 3:** Neither conventional nor micronised naringin nor naringenin causes adverse effects on body temperature or locomotor activity in mice. Treatment with vehicle (10 mL/kg, p.o.), conventional or micronised naringin or naringenin (30–300 mg/kg, p.o.) did not alter rectal body temperature, open-field locomotor activity, or rotarod performance. Data are presented as mean ± SEM or median and interquartile range (n = 6 animals; n = 5 for conventional naringenin (100 mg/kg, p.o.) due to video-recording failure for distance and speed travelled). No significant differences were observed (non-parametric data from the rotarod were analysed using the Kruskal–Wallis test followed by Dunn’s test, while other parametric data were analysed by one-way ANOVA followed by Bonferroni’s post hoc test).

Treatment	Δ Body Temperature	Distance Travelled (m)	Speed Travelled (m/s)	Falls in the Rotarod (nº)
Vehicle (10 mL/kg, p.o.)	0.15 ± 0.34	24.59 ± 3.65	0.0820 ± 0.0121	0.0 (0.0–2.0)
Naringin (30 mg/kg, p.o.)	−0.27 ± 0.29	22.40 ± 6.05	0.0748 ± 0.0201	0.0 (0.0–1.25)
Micronised Naringin (30 mg/kg, p.o.)	−0.90 ± 0.54	23.36 ± 2.33	0.0776 ± 0.00780	0.0 (0.0–0.5)
Naringin (100 mg/kg, p.o.)	−0.08 ± 0.11	15.17 ± 4.55	0.0505 ± 0.0151	0.0 (0.0–2.0)
Micronised Naringin (100 mg/kg, p.o.)	−0.23 ± 0.33	28.35 ± 3.22	0.0945 ± 0.0107	0.0 (0.0–0.0)
Naringin (300 mg/kg, p.o.)	−0.05 ± 0.35	31.01 ± 4.79	0.1035 ± 0.01597	0.0 (0.0–0.0)
Micronised Naringin (300 mg/kg, p.o.)	0.17 ± 0.26	22.11 ± 5.21	0.07367 ± 0.0173	0.0 (0.0–0.0)
Naringenin (30 mg/kg, p.o.)	−0.33 ± 0.58	24.94 ± 6.51	0.0830 ± 0.0217	0.5 (0.0–1.0)
Micronised Naringenin (30 mg/kg, p.o.)	0.45 ± 0.49	30.15 ± 2.59	0.09500 ± 0.0061	0.0 (0.0–1.25)
Naringenin (100 mg/kg, p.o.)	−0.08 ± 0.11	37.11 ± 4.63	0.1238 ± 0.01548	0.0 (0.0–2.0)
Micronised Naringenin (100 mg/kg, p.o.)	−0.32 ± 0.47	36.74 ± 6.26	0.1225 ± 0.02087	0.0 (0.0–0.0)
Naringenin (300 mg/kg, p.o.)	−0.05 ± 0.35	39.72 ± 4.35	0.1323 ± 0.01441	0.0 (0.0–0.0)
Micronised Naringenin (300 mg/kg, p.o.)	−0.22 ± 0.30	34.81 ± 4.40	0.1162 ± 0.01465	0.0 (0.0–0.0)

## Data Availability

The original contributions presented in this study are included in the article/Supplementary Materials. Further inquiries can be directed to the corresponding author.

## Supplementary Materials

The following supporting information can be downloaded at https://www.mdpi.com/article/10.3390/pharmaceutics18060747/s1: Figure S1. Gene stability classification. File S1: Data availability document.

## Abbreviations

The following abbreviations are used in this manuscript: AMPK—AMP-Activated Protein Kinase; ANOVA—Analysis of Variance; ATR-Attenuated Total Reflectance; AUC—Area Under the Curve; B1—Baseline 1; B2—Baseline 2; cDNA—Complementary Deoxyribonucleic Acid; CFA—Complete Freund’s Adjuvant; CGRP—Calcitonin Gene-Related Peptide; cm—Centimeter; CO_2_—Carbon Dioxide; Ct—Cycle Threshold; ΔΔCt—Comparative Cycle Threshold Method; DMSO—Dimethyl Sulfoxide; DNA—Deoxyribonucleic Acid; DRG—Dorsal Root Ganglion; DSC—Differential Scanning Calorimetry; F.E.B.—Free Energy of Binding; FTIR—Fourier Transform Infrared Spectroscopy; g—Gram; GAS—Gas Anti-Solvent; Gapdh—Glyceraldehyde-3-Phosphate Dehydrogenase; HCl—Hydrochloric Acid; HEK293—Human Embryonic Kidney 293 cells; HSD—Honestly Significant Difference; IB-4—Isolectin B4; i.p.—Intraperitoneal; i.pl.—Intraplantar; Kd—Dissociation constant; kg—kilogram; Ki—Inhibition Constant; LSD—Least Significant Difference; mM—millimolar; mg—milligram; mL—milliliter; mRNA—Messenger Ribonucleic Acid; NaCl—Sodium Chloride; NF-κB—Nuclear Factor kappa B; nm—Nanometer; nmol—Nanomole; PAR2—Protease-Activated Receptor 2; PBS—Phosphate-Buffered Saline; PCR—Polymerase Chain Reaction; PDB—Protein Data Bank; PGC-1α—Peroxisome Proliferator-Activated Receptor Gamma Coactivator 1-alpha; pH—Potential of Hydrogen; p.o.—Per Os (oral administration); PTFE—Polytetrafluoroethylene; PWT—Paw Withdrawal Threshold; qRT-PCR—Quantitative Real-Time Polymerase Chain Reaction; RQ—Relative Quantification; RMSD—Root Mean Square Deviation; RNA—Ribonucleic Acid; rpm—Revolutions Per Minute; s—seconds; SEM (microscopy)—Scanning Electron Microscopy; SEM (statistics)—Standard Error of the Mean; TNF-α—Tumor Necrosis Factor alpha; TRPA1—Transient Receptor Potential Ankyrin 1; TRPV1—Transient Receptor Potential Vanilloid 1; uPA—Urokinase-type Plasminogen Activator; UK—United Kingdom; UV-Vis—Ultraviolet–Visible; XRD—X-ray Diffraction; μg—microgram; μL—microliter; μm—micrometer.
