# Supplementary material for: Supercritical CO2 Antisolvent-Micronised Naringin and Naringenin Alleviate Paclitaxel-Induced Pain Syndrome
Source: Pharmaceutics. 2026 Jun 17;18(6):747. doi: 10.3390/pharmaceutics18060747 (PMC13307101; doi:10.3390/pharmaceutics18060747)
Supplement: Supplementary file 1 [file pharmaceutics-18-00747-s001.zip › File S1. Data availability document.pdf]

Supercritical CO_2 Antisolvent-Micronised Naringin and Naringenin Alleviate Paclitaxel-Induced Pain Syndrome

Gabriela Adriany Lisboa Zilli [1,2], Samara Cristina Mazon [1,2], Patricia Viera de Oliveira [3], Felipe Zaniol [3], Eulália Lopes da Silva Barros [3], Ângela Maria Lodi [2], Chaiane Lunelli Saretto [2], Hemyly Cardoso [2], Ana Lúcia Anversa Segatto [4], Sara Marchesan Oliveira [4], J. Vladimir Oliveira [1,3] and Indiara Brusco [1,2,*]

1 Graduate Program in Environmental Sciences, Community University of Chapecó Region—Unochapecó, Chapecó 89809-900, SC, Brazil; samaramazon2507@gmail.com

2 Behavioural and Neurochemistry Pharmacology of Pain Research Group, Community University of Chapecó Region—Unochapecó, Chapecó 89809-900, SC, Brazil;

3 Department of Chemical and Food Engineering, Federal University of Santa Catarina —UFSC, Florianópolis 88040-900, SC, Brazil; felipezaniol2@gmail.com (F.Z.); barroseulalialopes@gmail.com (E.L.d.S.B.)

4 Graduate Program in Biological Sciences: Toxicological Biochemistry, Centre of Natural and Exact Sciences, Federal University of Santa Maria—UFSM, Santa Maria 97105-900, RS, Brazil; analuciasegatto@gmail.com

* Correspondence: indiara.brusco@unochapeco.edu.br or indi_brusco@hotmail.com

Data Availability Statement:

Figure 5 – Dissolution rate

	Conventional Naringenin (PBS pH 6.8)					Micronised Naringenin (PBS pH 6.8)				
Time (min)	rep 1 (µg/mL)	rep 2 (µg/mL)	rep 3 (µg/mL)	Mean	SD	rep 1 (µg/mL)	rep 2 (µg/mL)	rep 3 (µg/mL)	Mean	SD
5	11,74784748	15,21648216	12,03075031	12,99836	1,926151	15,96678967	15,33948339	17,89790898	16,40139	1,333434
10	14,47847478	16,69249692	14,40467405	15,19188	1,300094	17,94710947	16,88929889	18,83271833	17,88971	0,97298
15	15,00738007	17,54120541	15,00738007	15,85199	1,462905	19,42312423	18,15621156	20,70233702	19,42722	1,273068
20	16,11439114	18,0701107	15,62238622	16,6023	1,29475	19,84132841	19,26322263	21,08364084	20,06273	0,930185
25	16,5202952	18,40221402	15,91758918	16,9467	1,296035	20,91143911	19,74292743	22,12915129	20,92784	1,193196
30	17,03690037	19,12792128	16,79089791	17,65191	1,284171	21,23124231	20,2595326	21,77244772	21,08774	0,766598
40	17,55350554	19,57072571	17,06150062	18,06191	1,329628	20,88683887	20,6900369	22,35055351	21,30914	0,90724
50	17,27060271	19,7798278	17,66420664	18,23821	1,349505	20,48093481	20,92373924	22,35055351	21,25174	0,977015
60	18,15621156	19,48462485	17,66420664	18,43501	0,941689	21,0098401	22,10455105	22,35055351	21,82165	0,713725
90	18,52521525	20,17343173	17,83640836	18,84502	1,200885	21,40344403	21,66174662	23,21156212	22,09225	0,977918
120	18,90651907	20,33333333	19,00492005	19,41492	0,796886	22,2398524	21,29274293	22,73185732	22,08815	0,731452
180	19,22632226	19,73062731	19,84132841	19,59943	0,327824	22,30135301	22,84255843	23,82656827	22,99016	0,773246

	Commercial Naringenin (HCl pH 1.2)					Micronised Naringenin (HCl pH 1.2)				
Time (min)	rep 1 (μg/mL)	rep 2 (μg/mL)	rep 3 (μg/mL)	Mean	SD	rep 1 (μg/mL)	rep 2 (μg/mL)	rep 3 (μg/mL)	Mean	SD
5	5,105781058	6,38499385	7,86100861	6,450595	1,378785	10,23493235	10,71463715	13,07626076	11,34194	1,520994
10	10,01353014	10,86223862	12,26445264	11,04674	1,136747	13,02706027	13,34686347	15,00738007	13,79377	1,063113
15	11,68634686	12,20295203	13,23616236	12,37515	0,789127	14,03567036	14,10947109	15,65928659	14,60148	0,916834
20	12,2398524	12,8302583	13,91266913	12,99426	0,848382	14,45387454	14,45387454	15,74538745	14,88438	0,745655
25	12,78105781	13,44526445	14,02337023	13,41656	0,621653	14,82287823	14,76137761	16,21279213	15,26568	0,820797
30	13,07626076	13,66666667	14,28167282	13,67487	0,602748	15,2902829	15,16728167	16,3603936	15,60599	0,656224
40	13,53136531	13,7896679	14,3800738	13,90037	0,435049	15,73308733	15,70848708	16,64329643	16,02829	0,532753
50	13,35916359	13,92496925	14,07257073	13,78557	0,376579	15,48708487	16,200492	16,6801968	16,12259	0,600359
60	13,71586716	14,07257073	14,699877	14,16277	0,498168	15,91758918	16,37269373	16,79089791	16,36039	0,436784
90	14,28167282	14,25707257	14,68757688	14,40877	0,241763	16,07749077	16,79089791	16,95079951	16,6064	0,464971
120	14,15867159	14,699877	15,03198032	14,63018	0,440807	16,88929889	16,66789668	17,09840098	16,8852	0,215281
180	14,28167282	15,16728167	15,19188192	14,88028	0,518554	16,79089791	16,95079951	16,91389914	16,8852	0,083725

	Commerical Naringin (PBS pH 6.8)					Micronised Naringin (PBS pH 6.8)				
Time (min)	rep 1 (μg/mL)	rep 2 (μg/mL)	rep 3 (μg/mL)	Mean	SD	rep 1 (μg/mL)	rep 2 (μg/mL)	rep 3 (μg/mL)	Mean	SD
5	66,30545817	50,28952191	62,36123506	59,65207	8,344593	76,18593625	80,4887251	79,49270916	78,72246	2,252435
10	69,57239044	55,38912351	65,82737052	63,59629	7,350139	79,29350598	84,87119522	81,04649402	81,73707	2,852248
15	72,75964143	58,77557769	69,33334661	66,95619	7,288804	80,20984064	86,94290837	82,87916335	83,34397	3,390514
20	74,23374502	59,45286853	69,49270916	67,72644	7,547076	81,24569721	84,63215139	82,4010757	82,75964	1,721466
25	75,26960159	61,24569721	73,87517928	70,13016	7,725694	83,51661355	88,29749004	78,69589641	83,50333	4,800811
30	78,5763745	64,23374502	73,39709163	72,06907	7,262953	81,04649402	90,01063745	83,71581673	84,92432	4,602643
40	81,48474104	65,42896414	79,49270916	75,4688	8,75162	79,77159363	86,50466135	83,07836653	83,11821	3,366711
50	83,11820717	89,13414343	79,33334661	83,8619	4,942541	81,68394422	88,97478088	83,11820717	84,59231	3,862488
60	85,86721116	78,85525896	82,04250996	82,25499	3,510802	81,32537849	88,93494024	82,87916335	84,37983	4,020616
90	88,8950996	81,48474104	86,62418327	85,66801	3,796585	80,72776892	89,37318725	80,40904382	83,50333	5,08594
120	91,52458167	85,66800797	89,29350598	88,8287	2,955824	79,97079681	86,86322709	81,20585657	82,67996	3,675069
180	90,09031873	90,28952191	91,08633466	90,48873	0,527042	79,17398406	81,28553785	84,67199203	81,7105	2,77353

	Commercial Naringin (HCl pH 1.2)					Micronised Naringin (HCl pH 1.2)				
Time (min)	rep 1 (μg/mL)	rep 2 (μg/mL)	rep 3 (μg/mL)	Mean	SD	rep 1 (μg/mL)	rep 2 (μg/mL)	rep 3 (μg/mL)	Mean	SD
5	55,03055777	47,34131474	63,71581673	55,36256	8,192298	78,37717131	94,07438247	87,58035857	86,6773	7,887474
10	59,41302789	54,79151394	67,73972112	60,64809	6,561863	85,42896414	94,83135458	86,42498008	88,8951	5,165013
15	61,92298805	58,09828685	72,16203187	64,0611	7,27158	85,34928287	93,15804781	88,93494024	89,14742	3,908716
20	63,87517928	61,1261753	75,15007968	66,71714	7,431356	82,75964143	94,99071713	86,86322709	88,20453	6,224879
25	65,70784861	63,9947012	78,29749004	69,33335	7,810289	87,22179283	92,87916335	91,32537849	90,47544	2,922884
30	67,85924303	66,6640239	82,00266932	72,17531	8,531697	86,3452988	94,51262948	89,17398406	90,01064	4,147447
40	69,5325498	69,29350598	84,43294821	74,41967	8,672579	86,14609562	93,39709163	90,56840637	90,0372	3,654569
50	69,73175299	70,8074502	86,06641434	75,53521	9,13614	83,75565737	94,47278884	90,17	89,46615	5,393124
60	71,56442231	71,68394422	87,46083665	76,90307	9,143491	85,46880478	95,03055777	89,37318725	89,95752	4,807584
90	77,06243028	77,46083665	91,20585657	81,90971	8,053165	83,59629482	91,52458167	88,65605578	87,92564	4,014294
120	79,93095618	82,95884462	93,6759761	85,52193	7,222079	86,94290837	90,92697211	88,13812749	88,66934	2,044463
180	82,64011952	88,01860558	97,22179283	89,29351	7,373963	87,58035857	93,43693227	90,24968127	90,42232	2,932101

Figure 7 – Capsaicin-induced nociception

Fig. 7A – Conventional Naringin

Group A	Group B	Group C	Group D	Group E
Vehicle+Vehicle	Vehicle+ Caps	NAR COM 30	NAR COM 100	NAR COM 300
9.8	56.0	101.0	49	30
0.0	44.0	28.6	51	37
0.0	72.0	107.0	23	35
0.0	88.0	111.0	44	48
0.0	63.0	87.0	53	28
2.0	120.0	79.0	57	64

Fig. 7B – Micronised Naringin

Group A	Group B	Group C	Group D	Group E
Vehicle+ Vehicle	Vehicle+Cap	NAR MICRO 30	NAR MICRO 100	NAR MIC 300
9.8	56.0	62	97	36
0.0	44.0	41	52	36
0.0	72.0	61	40	30
0.0	88.0	49	34	38
0.0	63.0	56	40	32
2.0	120.0	28	36	41

Fig. 7 C- Conventional Naringenin:

Group A	Group B	Group C	Group D	Group E
Vehicle+Vehicle	vehicle + Caps	NGN CON 30	NGN CON 100	NGN CON 300
9.8	56.0	35	36	57
0.0	58.0	50	35	28
0.0	44.0	40	36	56
0.0	48.0	59	62	61
0.0	88.0	59	80	18
0.0	63.0	57	20	10

Fig. 7 D- Micronised Naringenin:

Group A	Group B	Group C	Group D	Group E
Vehicle+Vehicle	Vehicle+Caps	NGN MICRO 30	NGN MICRO 100	NGN MICRO 300
9.8	56.0	49	40	29
0.0	58.0	40	32	40
0.0	44.0	50	67	30
0.0	48.0	42	24	55
0.0	88.0	45	8	53
0.0	63.0	53	21	6

<u>**Table 3 – Adverse effects on body temperature and locomotor activity**</u>

Δ Body temperature

Group A	Group B	Group C	Group D	Group E	Group F	Group G	Group H	Group I	Group J	Group K	Group L	Group M
Vehicle	NAR MICRO 30	NAR COM 30	NAR MICRO 100	NAR COM 100	NAR COM 300	NAR MICRO 300	NGN COM 30	NGN MICRO 30	NGN COM 100	NGN MICRO 100	NGN COM 300	NGN MICRO 300
-0.4	0.4	-0.8	0.5	-0.2	-0.9	0.2	2.0	2.8	-0.2	-0.4	-0.9	-0.7
-0.4	-0.3	-1.0	0.3	0.4	1.0	1.0	0.6	0.4	0.4	0.3	1.0	-0.7
-0.9	-0.1	0.9	-1.0	0.0	1.0	0.8	-1.4	0.2	0.0	-1.9	1.0	1.1
1.2	-1.4	0.2	-1.5	-0.2	-0.9	-0.2	-0.8	-0.6	-0.2	-1.3	-0.9	-0.7
0.9	-0.7	-0.4	0.0	-0.1	-0.4	-0.1	-0.5	-0.1	-0.1	1.3	-0.4	-0.6
0.5	-3.3	-0.5	0.3	-0.4	-0.1	-0.7	-1.9	0.0	-0.4	0.1	-0.1	0.3

Distance travelled (m)

* Group J n= 5 due to video recording failure

Group A	Group B	Group C	Group D	Group E	Group F	Group G	Group H	Group I	Group J	Group K	Group L	Group M
Vehicle	NAR MICRO 30	NAR COM 30	NAR MICRO 100	NAR COM 100	NAR MICRO 300	NAR COM 300	NGN COM 30	NGN MICRO 30	NGN COM 100	NGN MICRO 100	NGN COM 300	NGN MICRO 300
32.41	25.25	33.22	39.71	22.58	27.26	50.98	28.25	28.98	28.76	30.74	26.45	37.24
26.43	19.64	45.24	30.71	18.53	26.90	36.50	34.02	28.08	36.53	56.87	51.35	36.50
25.99	22.81	14.30	29.88	3.43	40.21	29.39	1.89	35.93	30.95	44.81	29.58	22.96
12.92	18.95	23.47	30.45	32.19	3.58	21.82	17.55	25.80	34.47	10.98	49.94	29.04
34.84	33.93	13.35	22.76	6.88	12.37	29.50	19.32	39.45	54.82	38.66	35.57	53.97
14.96	19.58	4.82	16.61	7.43	22.33	17.87	48.60	22.63		38.36	45.41	29.16

Speed travelled (m/s)

*Group J n= 5 due to video recording failure

Group A	Group B	Group C	Group D	Group E	Group F	Group G	Group H	Group I	Group J	Group K	Group L	Group M
Vehicle	NAR MICRO 30	NAR COM 30	NAR MICRO 100	NAR COM 100	NAR MICRO 300	NAR COM 300	NGN COM 30	NGN MICRO 30	NGN COM 100	NGN MICRO 100	NGN COM 300	NGN MICRO 300
0.108	0.084	0.111	0.132	0.075	0.091	0.170	0.094	0.097	0.096	0.102	0.088	0.124
0.088	0.065	0.151	0.102	0.062	0.090	0.122	0.113	0.094	0.122	0.190	0.171	0.122
0.087	0.076	0.048	0.100	0.011	0.134	0.098	0.006	0.120	0.103	0.149	0.099	0.077
0.043	0.063	0.078	0.102	0.107	0.012	0.073	0.059	0.086	0.115	0.037	0.166	0.097
0.116	0.113	0.045	0.076	0.023	0.041	0.098	0.064	0.098	0.183	0.129	0.119	0.180
0.050	0.065	0.016	0.055	0.025	0.074	0.060	0.162	0.075		0.128	0.151	0.097

Falls in the rotarod (nº)

Group A	Group B	Group C	Group D	Group E	Group F	Group G	Group H	Group I	Group J	Group K	Group L	Group M
Vehicle	NAR MICRO 30	NAR COM 30	NAR MICRO 100	NAR COM 100	NAR COM 300	NAR Micro 300	NGN COM 30	NGN MICRO 30	NGN COM 100	NGN MICRO 100	NGN COM 300	NGN MICRO 300
2	0	0	0	2	0	0	0	0	2	0	0	0
0	0	0	0	0	0	0	0	1	0	0	0	0
0	0	1	0	2	0	0	1	0	2	0	0	0
2	0	0	0	0	0	0	0	2	0	0	0	0
0	2	0	0	0	0	0	1	0	0	0	0	0
0	0	2	0	0	0	0	1	0	0	0	0	0

Figure 8 – TRPV1 mRNA expression (PCR analyses)

Fig. 8A – Naringin results

* 0.000544250 = outlier according to Grubb's statistical test in GraphPad Prism

Group A	Group B	Group C	Group D
Veículo	Pacli	NAR	NAR micro
0.001624055	0.003159867	0.002956542	0.001475449
0.001060862	0.003449640	0.001475293	0.001598798
0.001782885	0.003441912	0.003479976	0.001912996
0.002371427	0.002459508	0.002133123	0.001283630
0.001206883	*0.000544250**	0.002939490	0.000588723
0.000276790	0.002277975	0.001841454	0.002135472

Fig. 8B – Naringenin results

* 0.000544250 = outlier according to Grubb's statistical test in GraphPad Prism

Group A	Group B	Group C	Group D
Veículo	Pacli	NGN	NGN micro
0.001624055	0.003159867	0.0014334480	0.001883175
0.001060862	0.003449640	0.0002773280	0.001680417
0.001782885	0.003441912	0.0000775345	0.002686948
0.002371427	0.002459508	0.0020214630	0.001150691
0.001206883	*0.000544250**	0.0019006220	0.000898737
0.000276790	0.002277975	0.0011744100	0.003760047

<u>Figure 9 – Paclitaxel-induced chronic pain model</u>

* Group C n = 5 due to limited animal availability from the breeding facility

Figure 9A – Mechanical allodynia – Naringin results

Table format: Grouped	Group A						Group B						Group C				
	Paclitaxel (1 mg/kg, i.p.) + NAR MICRO (100mg/kg, p.o.)						Paclitaxel (1 mg/kg, i.p.) + NAR COM (100 mg/kg, p.o.)						Paclitaxel (1 mg/kg, i.p.) + Vehicle (10ml/kg,				
	A:1	A:2	A:3	A:4	A:5	A:6	B:1	B:2	B:3	B:4	B:5	B:6	C:1	C:2	C:3	C:4	C:5
B1	3.105600	3.105600	3.105600	2.093100	3.105600	1.166200	2.093100	4.796100	3.105600	3.105600	1.680600	3.105600	3.105600	3.105600	1.680600	3.105600	3.105600
B2	0.570100	0.566200	0.392900	0.274700	0.566200	0.570100	0.192100	0.392900	0.392900	0.566200	0.566200	0.566200	0.566200	0.570100	0.566200	0.309700	0.392900
0.5	0.566194	1.166225	0.815413	0.815413	0.570129	1.016613	0.392893	0.815413	0.392893	0.392893	0.570129	0.815413	0.392893	0.392893	0.570129	0.570129	0.570129
1	1.166226	2.093062	2.093062	2.093062	2.093062	0.566194	1.166226	1.433696	0.570130	0.566194	0.809785	1.166226	0.570130	0.570130	0.566194	0.570130	0.570130
2	1.680636	3.105629	1.166226	1.166226	2.093062	0.815414	1.166226	0.566194	1.166226	1.166226	1.166226	0.566194	0.566194	0.570130	0.395624	0.570130	0.274707
4	1.433695	2.093061	0.815413	2.093061	1.680635	0.815413	1.166225	0.570129	0.815413	0.392893	0.815413	0.163850	0.566194	0.566194	0.392893	0.309730	1.016613
6	0.815413	0.809785	1.433695	0.815413	0.815413	0.570129	0.570129	0.566194	0.566194	1.166225	0.815413	1.166225	0.815413	0.570129	0.570129	0.392893	0.570129

Figure 9B – Mechanical allodynia – AUC- Naringin results

Group A	Group B	Group C
Pacli + vehicle	Pacli + Nar convencional	Pacli + Nar Micronized
2.307	3.980	4.97
2.188	3.261	8.28
1.925	3.031	5.20
1.932	2.905	6.17
2.432	3.484	6.56
	2.887	2.99

Figure 9C – Mechanical allodynia – Naringenin results

Table format: Grouped	Group A						Group B						Group C				
	Paclitaxel (1 mg/kg, i.p.) + NGN MIC (100 mg/kg, p.o.)						Paclitaxel (1 mg/kg, i.p.) + NGN COM (100 mg/kg, p.o.)						Paclitaxel (1 mg/kg, i.p.) + Vehicle (10ml/kg,				
	A:1	A:2	A:3	A:4	A:5	A:6	B:1	B:2	B:3	B:4	B:5	B:6	C:1	C:2	C:3	C:4	C:5
B1	1.680600	3.105600	2.093100	3.105600	3.105600	3.105600	2.093100	3.105600	3.105600	3.105600	1.433700	2.093100	3.105600	3.105600	1.680600	3.105600	3.105600
B2	0.392900	0.570100	0.392900	0.566200	0.815400	0.392900	0.392900	0.815400	0.570100	0.570100	0.274700	0.392900	0.566200	0.570100	0.566200	0.309700	0.392900
0.5	0.815413	0.570129	0.566194	1.166225	0.809785	0.392893	0.570129	0.809785	0.815413	0.274707	0.570129	0.392893	0.392893	0.392893	0.570129	0.570129	0.570129
1	1.166226	1.680636	0.815414	0.815414	1.166226	1.166226	0.566194	0.815414	0.815414	0.570130	0.570130	0.815414	0.570130	0.570130	0.566194	0.570130	0.570130
2	1.166226	0.566194	1.433696	2.093062	0.815414	1.433696	0.392893	0.815414	0.815414	0.570130	0.395624	0.570130	0.065158	0.570130	0.395624	0.570130	0.274707
4	1.166225	0.570129	1.166225	0.093190	0.809785	0.815413	0.065157	0.570129	0.570129	0.570129	0.570129	0.570129	0.566194	0.566194	0.392893	0.309730	1.016613
6	1.016613	0.570129	0.566194	0.570129	0.570129	0.566194	0.566194	0.566194	0.570129	0.220339	0.570129	0.392893	0.815413	0.570129	0.570129	0.392893	0.570129

Figure 9D – Mechanical allodynia – AUC - Naringenin results

Group A	Group B	Group C
Pacli + vehicle	Pacli + NGN convencional	Pacli + NGN Micronized
1.806	1.592	4.42
2.188	2.889	3.39
1.925	2.894	3.98
1.932	1.958	3.87
2.432	2.106	3.48
	2.349	3.90

Figure 9E – Cold allodynia – Naringin results

Table format: Grouped	Group A						Group B						Group C				
	Paclitaxel (1 mg/kg, i.p.) + NAR MIC (100 mg/kg, p.o.)						Paclitaxel (1 mg/kg, i.p.) + NAR COM (100 mg/kg, p.o.)						Paclitaxel (1 mg/kg, i.p.) + Vehicle (10ml/kg				
	A:1	A:2	A:3	A:4	A:5	A:6	B:1	B:2	B:3	B:4	B:5	B:6	C:1	C:2	C:3	C:4	C:5
B1	2.0000	2.0000	1.0000	3.0000	1.0000	2.0000	0.0000	0.0000	3.0000	0.0000	4.0000	1.0000	2.0000	0.0000	0.0000	0.0000	1.0000
B2	7.0000	10.0000	5.0000	5.0000	6.0000	7.0000	6.0000	7.0000	9.0000	8.0000	5.0000	8.0000	8.0000	8.0000	6.0000	6.0000	6.0000
1	2.0000	4.0000	5.0000	3.0000	2.0000	6.0000	3.0000	5.0000	7.0000	3.0000	5.0000	9.0000	8.0000	7.0000	6.0000	6.0000	6.0000
2	2.0000	2.0000	2.0000	2.0000	1.0000	8.0000	0.0000	2.0000	6.0000	5.0000	1.0000	7.0000	8.0000	7.0000	6.0000	6.0000	6.0000
4	5.0000	2.0000	6.0000	6.0000	1.0000	8.0000	3.0000	3.0000	4.0000	5.0000	4.0000	5.0000	7.0000	7.0000	6.0000	4.0000	7.0000
6	4.0000	3.0000	5.0000	3.0000	4.0000	5.0000	3.0000	6.0000	6.0000	5.0000	6.0000	8.0000	6.0000	5.0000	7.0000	5.0000	6.0000
8	6.0000	5.0000	6.0000	6.0000	3.0000	8.0000	5.0000	5.0000	6.0000	4.0000	6.0000	5.0000	5.0000	6.0000	5.0000	5.0000	6.0000

Figure 9F – Cold allodynia - AUC - Naringin results

Group A	Group B	Group C
Pacli + vehicle	Pacli + Nar convencional	Pacli + Nar Micronized
27.500	10.000	15.00
25.500	16.000	11.50
24.500	22.500	18.50
20.500	18.500	15.50
25.000	16.500	8.50
	27.000	28.00

Figure 9G – Cold allodynia – Naringenin results

Table format: Grouped	Group A						Group B						Group C				
	Paclitaxel (1 mg/kg, i.p.) + NGN MIC (100 mg/kg, p.o.)						Paclitaxel (1 mg/kg, i.p.) + NGN COM (100 mg/kg, p.o.)						Paclitaxel (1 mg/kg, i.p.) + Vehicle (10ml/k				
	A:1	A:2	A:3	A:4	A:5	A:6	B:1	B:2	B:3	B:4	B:5	B:6	C:1	C:2	C:3	C:4	C:5
B1	0.0000	1.0000	4.0000	0.0000	0.0000	1.0000	1.0000	0.0000	0.0000	0.0000	2.0000	0.0000	2.0000	0.0000	0.0000	0.0000	1.0000
B2	7.0000	7.0000	7.0000	6.0000	6.0000	6.0000	5.0000	6.0000	6.0000	6.0000	7.0000	8.0000	8.0000	8.0000	6.0000	6.0000	6.0000
1	3.0000	3.0000	3.0000	5.0000	5.0000	5.0000	3.0000	5.0000	5.0000	6.0000	3.0000	5.0000	8.0000	7.0000	6.0000	6.0000	6.0000
2	6.0000	5.0000	1.0000	2.0000	4.0000	4.0000	3.0000	7.0000	1.0000	6.0000	5.0000	6.0000	8.0000	7.0000	6.0000	6.0000	6.0000
4	7.0000	8.0000	5.0000	6.0000	4.0000	5.0000	6.0000	5.0000	5.0000	5.0000	5.0000	6.0000	7.0000	7.0000	6.0000	4.0000	7.0000
6	7.0000	8.0000	4.0000	3.0000	4.0000	4.0000	3.0000	4.0000	4.0000	5.0000	4.0000	6.0000	6.0000	5.0000	7.0000	5.0000	6.0000
8	7.0000	7.0000	4.0000	5.0000	5.0000	2.0000	5.0000	3.0000	4.0000	6.0000	5.0000	6.0000	5.0000	6.0000	5.0000	5.0000	6.0000

Figure 9H – Cold allodynia - AUC - Naringenin results

Group A	Group B	Group C
Pacli + vehicle	Pacli + NGN convencional	Pacli + NGN Micronized
27.500	16.000	25.00
25.500	20.000	26.00
24.500	14.500	13.50
20.500	22.000	16.00
25.000	18.000	17.00
	23.500	16.50

Figure 10 – Paclitaxel-induced acute pain model

Figure 10 A – Mechanical allodynia – Naringin results

Table format: Grouped	Group A Paclitaxel (1 mg/kg, i.p.) + Veículo (10ml/kg, v.o.)						Group B Paclitaxel (1 mg/kg, i.p.) + NAR COM (100 mg/kg, v.o.)						Group C Paclitaxel (1 mg/kg, i.p.) + NAR MIC (100 mg/kg, v.o.)					
	A:1	A:2	A:3	A:4	A:5	A:6	B:1	B:2	B:3	B:4	B:5	B:6	C:1	C:2	C:3	C:4	C:5	C:6
B1	2.0931	0.8154	3.1056	3.1056	3.1056	1.6806	3.1056	3.1056	2.0931	2.0931	2.0931	3.1056	3.1056	2.0931	3.1056	3.1056	3.1056	3.1056
B2	0.5662	0.3929	0.5701	0.2747	0.5701	0.2747	0.2747	0.3929	0.2747	0.5701	0.5662	0.5701	0.5662	0.5662	0.3929	0.3929	0.3929	0.2747
0.5	0.3929	0.5662	0.3929	0.1333	0.5701	0.5701	0.3929	0.5701	0.2203	0.5662	0.8154	0.3956	0.8154	0.5701	0.5662	0.8154	0.8154	0.5701
1	0.5662	0.5662	0.3929	0.5662	0.3929	0.5701	0.5662	0.5701	0.8154	0.8154	0.5701	0.5662	1.1662	0.8154	0.8154	1.4337	1.4337	0.8154
2	0.5701	0.8154	0.3929	0.2747	0.3929	0.5662	0.3929	0.5701	0.5701	0.8154	0.8154	0.8154	2.0931	2.0931	0.8154	2.0931	2.0931	0.8154
4	0.3929	0.5707	0.3929	0.1527	0.3929	0.3929	0.5701	0.3956	0.8098	0.5701	0.8154	0.5662	1.1662	0.8154	0.8154	1.1662	1.6806	0.8154
6	0.5701	0.5662	0.5662	0.3956	0.2747	0.3929	0.1921	0.5662	0.5701	0.3956	0.5701	0.5701	0.8154	0.8154	0.3929	0.5662	0.8154	0.5701
8	0.5701	0.5701	0.5701	0.3097	0.3097	0.3956	0.2766	0.1921	0.5662	0.5701	0.5662	0.5701	0.5701	0.5662	0.2747	0.5701	0.8154	0.5701
Title																		

Figure 10 B – Mechanical allodynia – AUC- Naringin results

Group A	Group B	Group C
Pacli + Vehi	Nar CON	Nar MICRO
2.581	2.056	5.934
3.087	2.483	5.107
2.226	3.159	3.260
1.611	3.165	5.952
1.893	3.462	6.838
2.405	3.001	3.586

Figure 10 C – Mechanical allodynia – Naringenin results

Table format: Grouped	Group A Paclitaxel (1 mg/kg, i.p.) + Veículo (10ml/kg, v.o.)						Group B Paclitaxel (1 mg/kg, i.p.) + NGN COM (100 mg/kg, v.o.)						Group C Paclitaxel (1 mg/kg, i.p.) + NGN MIC (100 mg/kg, v.o.)					
	A:1	A:2	A:3	A:4	A:5	A:6	B:1	B:2	B:3	B:4	B:5	B:6	C:1	C:2	C:3	C:4	C:5	C:6
B1	2.0931	0.8154	3.1056	3.1056	3.1056	1.6806	3.105600	1.166200	2.093100	3.105600	3.105600	3.105600	3.105600	2.093100	3.105600	2.093100	3.105600	2.093100
B2	0.5662	0.3929	0.5701	0.2747	0.5701	0.2747	0.570100	0.392900	0.566200	0.392900	0.274700	0.566200	0.274700	0.192100	0.392900	0.566200	0.566200	0.566200
0.5	0.3929	0.5662	0.3929	0.1333	0.5701	0.5701	0.815400	0.570100	1.166200	0.570100	0.815400	0.566200	0.815400	1.166200	0.815400	0.815400	1.166200	0.815400
1	0.5662	0.5662	0.3929	0.5662	0.3929	0.5701	0.815400	0.815400	1.166200	0.815400	0.566200	0.570100	0.815400	1.680600	1.680600	2.093100	2.093100	0.815400
2	0.5701	0.8154	0.3929	0.2747	0.3929	0.5662	0.815400	0.570100	0.570100	0.570100	0.815400	0.815400	0.815400	1.166200	1.166200	1.166200	1.166200	0.815400
4	0.3929	0.5707	0.3929	0.1527	0.3929	0.3929	0.815400	0.570100	0.815400	0.566200	0.815400	0.570100	0.815400	0.566200	1.433700	0.815400	1.433700	0.815400
6	0.5701	0.5662	0.5662	0.3956	0.2747	0.3929	0.570100	0.570100	0.395600	0.570100	0.815400	0.566200	0.570100	1.433700	1.166200	0.815400	0.566200	0.815400
8	0.5701	0.5701	0.5701	0.3097	0.3097	0.3956	0.570100	0.395600	0.570100	0.570100	0.566200	0.815400	0.192100	0.570100	0.274700	0.220300	0.570100	0.815400

Figure 10 D – Mechanical allodynia – AUC - Naringenin results

Group A	Group B	Group C
Pacli + Vehi	NGN CON	NGN MICRO
2.581	3.709	3.520
3.087	3.009	5.715
2.226	3.815	5.992
1.611	3.092	5.408
1.893	3.703	6.127
2.405	3.213	4.077

Figure 10 E – Cold allodynia – Naringin results

Table format: Grouped		Group A: Paclitaxel (1 mg/kg, i.p.) + Vehicle (10ml/kg, p.o.)						Group B: Paclittaxel (1 mg/kg, i.p.) + NAR COM (100 mg/kg, p.o.)						Group C: Paclittaxel (1 mg/kg, i.p.) + NAR MIC (100 mg/kg, p.o.)					
		A:1	A:2	A:3	A:4	A:5	A:6	B:1	B:2	B:3	B:4	B:5	B:6	C:1	C:2	C:3	C:4	C:5	C:6
1	B1	0.0000	1.0000	0.0000	1.0000	1.0000	2.0000	3.0000	0.0000	1.0000	1.0000	1.0000	2.0000	1.0000	1.0000	0.0000	1.0000	0.0000	0.0000
2	B2	4.0000	5.0000	6.0000	7.0000	5.0000	5.0000	6.0000	7.0000	5.0000	5.0000	8.0000	7.0000	5.0000	7.0000	6.0000	7.0000	5.0000	5.0000
3	1	4.0000	5.0000	6.0000	6.0000	5.0000	5.0000	2.0000	5.0000	3.0000	3.0000	0.0000	3.0000	1.0000	1.0000	5.0000	2.0000	0.0000	1.0000
4	2	5.0000	6.0000	5.0000	4.0000	7.0000	6.0000	3.0000	6.0000	4.0000	4.0000	2.0000	4.0000	2.0000	2.0000	6.0000	2.0000	2.0000	2.0000
5	4	5.0000	6.0000	5.0000	6.0000	4.0000	6.0000	2.0000	6.0000	4.0000	4.0000	3.0000	5.0000	3.0000	2.0000	4.0000	6.0000	0.0000	3.0000
6	6	5.0000	6.0000	7.0000	6.0000	5.0000	5.0000	7.0000	6.0000	3.0000	4.0000	4.0000	6.0000	4.0000	5.0000	4.0000	4.0000	3.0000	3.0000
7	8	6.0000	5.0000	5.0000	5.0000	4.0000	6.0000	4.0000	6.0000	4.0000	5.0000	5.0000	6.0000	5.0000	5.0000	4.0000	5.0000	4.0000	4.0000
8	Title																		

Figure 10 F – Cold allodynia - AUC - Naringin results

Group A	Group B	Group C
Pacli + Vehi	Nar CON	Nar MICRO
20.000	15.000	12.000
23.000	23.500	12.000
22.500	14.500	18.500
21.500	16.000	15.500
20.500	11.500	7.000
22.500	19.500	10.500

Figure 10 G – Cold allodynia – Naringenin results

Table format: Grouped		Group A: Paclitaxel (1 mg/kg, i.p.) + Vehicle (10ml/kg, p.o.)						Group B: aclittaxel (1 mg/kg, i.p.) + NGN COM (100 mg/kg, p.(						Group C: Paclitaxel (1 mg/kg, i.p.) + NGN MIC (100 mg/kg, p.o.)					
		A:1	A:2	A:3	A:4	A:5	A:6	B:1	B:2	B:3	B:4	B:5	B:6	C:1	C:2	C:3	C:4	C:5	C:6
1	B1	0.0000	1.0000	0.0000	1.0000	1.0000	2.0000	0.0000	2.0000	3.0000	3.0000	1.0000	1.0000	3.0000	3.0000	0.0000	0.0000	1.0000	0.0000
2	B2	4.0000	5.0000	6.0000	7.0000	5.0000	5.0000	5.0000	6.0000	4.0000	6.0000	5.0000	5.0000	6.0000	5.0000	5.0000	6.0000	4.0000	4.0000
3	1	4.0000	5.0000	6.0000	6.0000	5.0000	5.0000	4.0000	5.0000	6.0000	4.0000	4.0000	5.0000	2.0000	3.0000	1.0000	3.0000	3.0000	4.0000
4	2	5.0000	6.0000	5.0000	4.0000	7.0000	6.0000	4.0000	4.0000	6.0000	6.0000	2.0000	5.0000	6.0000	3.0000	3.0000	2.0000	3.0000	4.0000
5	4	5.0000	6.0000	5.0000	6.0000	4.0000	6.0000	3.0000	5.0000	3.0000	5.0000	4.0000	4.0000	3.0000	5.0000	4.0000	2.0000	4.0000	5.0000
6	6	5.0000	6.0000	7.0000	6.0000	5.0000	5.0000	5.0000	4.0000	4.0000	4.0000	3.0000	4.0000	6.0000	4.0000	5.0000	2.0000	3.0000	5.0000
7	8	6.0000	5.0000	5.0000	5.0000	4.0000	6.0000	4.0000	5.0000	5.0000	4.0000	4.0000	5.0000	6.0000	3.0000	3.0000	4.0000	6.0000	4.0000

Figure 10 H – Cold allodynia - AUC - Naringenin results

Group A	Group B	Group C
Pacli + Vehi	NGN CON	NGN MICRO
20.000	16.000	19.000
23.000	18.000	15.000
22.500	18.500	14.000
21.500	19.000	9.500
20.500	13.000	14.500
22.500	18.000	18.000

Figure 11 – Paclitaxel-induced heat hyperalgesia

Figure 11A – Acute

* 2 Group D and 1 Group E = outlier according to Grubb's statistical test in GraphPad Prism

Group A	Group B	Group C	Group D	Group E	Group F
Vehicle + Vehicle	Paclitaxel + Vehicle	Paclitaxel + NAR CON	Paclitaxel + NAR MICRO	Paclitaxel + NGN CON	Paclitaxel + NGN MICRO
25	7	11	30	25*	18
30	13	12	20*	14	30
21	10	21	30	13	29
30	11	9	29	13	24
28	12	18	30	15	24
30	17	17	30	15	30

Figure 11B – Chronic

Group A	Group B	Group C	Group D	Group E	Group F
Vehicle + Vehicle	Paclitaxel + Vehicle	Paclitaxel + NAR CON	Paclitaxel + NAR MICRO	Paclitaxel + NGN CON	Paclitaxel + NGN MICRO
30	10	30	28	13	15
30	10	21	21	10	30
16	15	12	27	13	17
16	15	13	22	20	17
30	16	20	24	16	14
28	13	18	18	11	25

Comparisons for capsaicin-induced paw licking

Outcome	Treatment	Test	F value	df	P value
**Paw licking (capsaicin)**	Naringin	One-way ANOVA	F = 15.89	(4, 25)	< 0.0001
	Micronised naringin	One-way ANOVA	F = 13.79	(4, 25)	< 0.0001
	Naringenin	One-way ANOVA	F = 10.99	(4, 25)	< 0.0001
	Micronised naringenin	One-way ANOVA	F = 13.60	(4, 25)	< 0.0001

Bonferroni's multiple comparisons test	Mean Diff.	95.00% CI of diff.	Significant?	Summary	Adjusted P Value
Vehicle+ Caps vs. Vehicle+Vehicle	71.87	40.75 to 103.0	Yes	****	<0.0001
Vehicle+ Caps vs. NAR COM 30	-11.77	-42.89 to 19.35	No	ns	>0.9999
Vehicle+ Caps vs. NAR COM 100	27.67	-3.454 to 58.79	No	ns	0.0982
Vehicle+ Caps vs. NAR COM 300	33.50	2.379 to 64.62	Yes	*	0.0309

Bonferroni's multiple comparisons test	Mean Diff.	95.00% CI of diff.	Significant?	Summary	Adjusted P Value
Vehicle+Cap vs. Vehicle+ Vehicle	71.87	44.87 to 98.87	Yes	****	<0.0001
Vehicle+Cap vs. NAR MICRO 30	24.33	-2.667 to 51.33	No	ns	0.0913
Vehicle+Cap vs. NAR MICRO 100	24.00	-3.001 to 51.00	No	ns	0.0983
Vehicle+Cap vs. NAR MIC 300	38.33	11.33 to 65.33	Yes	**	0.0031

Bonferroni's multiple comparisons test	Mean Diff.	95.00% CI of diff.	Significant?	Summary	Adjusted P Value
Vehicle+Cap vs. Vehicle+Vehicle	57.87	32.38 to 83.36	Yes	****	<0.0001
Vehicle+Cap vs. NGN CON 30	9.500	-15.99 to 34.99	No	ns	>0.9999
Vehicle+Cap vs. NGN CON 100	14.67	-10.82 to 40.16	No	ns	0.5360
Vehicle+Cap vs. NGN CON 300	21.17	-4.322 to 46.66	No	ns	0.1383

Bonferroni's multiple comparisons test	Mean Diff.	95.00% CI of diff.	Significant?	Summary	Adjusted P Value
Vehicle+Caps vs. Vehicle+Vehicle	57.87	35.64 to 80.10	Yes	****	<0.0001
Vehicle+Caps vs. NGN MICRO 30	13.00	-9.231 to 35.23	No	ns	0.5123
Vehicle+Caps vs. NGN MICRO 100	27.50	5.269 to 49.73	Yes	*	0.0108
Vehicle+Caps vs. NGN MICRO 300	24.00	1.769 to 46.23	Yes	*	0.0303

Comparisons for adverse effects

	Treatments	Test	F value	df	P value
**Body temperature variation (ΔT°)**	Vehicle, Naringin and Naringenin convetional or micronised	One-way ANOVA	F = 0.7209	(12, 65)	0.7258
**Distance travelled (open field)**	Vehicle, Naringin and Naringenin convetional or micronised	One-way ANOVA	F = 2.360	(12, 64)	0.0139
**Speed distance traveled (open field)**	Vehicle, Naringin and Naringenin convetional or micronised	One-way ANOVA	F = 2.387	(12, 64)	0.0129
**Number of falls (rota-rod)**	Vehicle, Naringin and Naringenin convetional or micronised	Kruskal-Wallis test	-	-	0.2000

Body temperature

Bonferroni's multiple comparisons	Mean Diff.	95.00% CI of diff.	Significant?	Summary	Adjusted P Value
Vehicle vs. NAR MICRO 30	1.050	-0.5309 to 2.631	No	ns	0.6335
Vehicle vs. NAR COM 30	0.4167	-1.164 to 1.998	No	ns	>0.9999
Vehicle vs. NAR MICRO 100	0.3833	-1.198 to 1.964	No	ns	>0.9999
Vehicle vs. NAR COM 100	0.2333	-1.348 to 1.814	No	ns	>0.9999
Vehicle vs. NAR COM 300	0.2000	-1.381 to 1.781	No	ns	>0.9999
Vehicle vs. NAR MICRO 300	-0.01667	-1.598 to 1.564	No	ns	>0.9999
Vehicle vs. NGN COM 30	0.4833	-1.098 to 2.064	No	ns	>0.9999
Vehicle vs. NGN MICRO 30	-0.3000	-1.881 to 1.281	No	ns	>0.9999
Vehicle vs. NGN COM 100	0.2333	-1.348 to 1.814	No	ns	>0.9999
Vehicle vs. NGN MICRO 100	0.4667	-1.114 to 2.048	No	ns	>0.9999
Vehicle vs. NGN COM 300	0.2000	-1.381 to 1.781	No	ns	>0.9999
Vehicle vs. NGN MICRO 300	0.3667	-1.214 to 1.948	No	ns	>0.9999

Distance travelled

Bonferroni's multiple comparisons test	Mean Diff.	95.00% CI of diff.	Significant?	Summary	Adjusted P Value
Vehicle vs. NAR MICRO 30	1.232	-18.34 to 20.80	No	ns	>0.9999
Vehicle vs. NAR COM 30	2.192	-17.38 to 21.76	No	ns	>0.9999
Vehicle vs. NAR MICRO 100	-3.762	-23.33 to 15.81	No	ns	>0.9999
Vehicle vs. NAR COM 100	9.418	-10.15 to 28.99	No	ns	>0.9999
Vehicle vs. NAR MICRO 300	2.483	-17.09 to 22.05	No	ns	>0.9999
Vehicle vs. NAR COM 300	-6.418	-25.99 to 13.15	No	ns	>0.9999
Vehicle vs. NGN COM 30	-0.3467	-19.92 to 19.22	No	ns	>0.9999
Vehicle vs. NGN MICRO 30	-5.553	-25.12 to 14.02	No	ns	>0.9999
Vehicle vs. NGN COM 100	-12.51	-33.04 to 8.011	No	ns	0.8961
Vehicle vs. NGN MICRO 100	-12.15	-31.71 to 7.425	No	ns	0.8371
Vehicle vs. NGN COM 300	-15.13	-34.69 to 4.445	No	ns	0.2989
Vehicle vs. NGN MICRO 300	-10.22	-29.79 to 9.350	No	ns	>0.9999

Speed travelled

Bonferroni's multiple comparisons test	Mean Diff.	95.00% CI of diff.	Significant?	Summary	Adjusted P Value
Vehicle vs. NAR MICRO 30	0.004333	-0.06047 to 0.06914	No	ns	>0.9999
Vehicle vs. NAR COM 30	0.007167	-0.05764 to 0.07197	No	ns	>0.9999
Vehicle vs. NAR MICRO 100	-0.01250	-0.07730 to 0.05230	No	ns	>0.9999
Vehicle vs. NAR COM 100	0.03150	-0.03330 to 0.09630	No	ns	>0.9999
Vehicle vs. NAR MICRO 300	0.008333	-0.05647 to 0.07314	No	ns	>0.9999
Vehicle vs. NAR COM 300	-0.02150	-0.08630 to 0.04330	No	ns	>0.9999
Vehicle vs. NGN COM 30	-0.001000	-0.06580 to 0.06380	No	ns	>0.9999
Vehicle vs. NGN MICRO 30	-0.01300	-0.07780 to 0.05180	No	ns	>0.9999
Vehicle vs. NGN COM 100	-0.04180	-0.1098 to 0.02617	No	ns	0.8671
Vehicle vs. NGN MICRO 100	-0.04050	-0.1053 to 0.02430	No	ns	0.8144
Vehicle vs. NGN COM 300	-0.05033	-0.1151 to 0.01447	No	ns	0.2907
Vehicle vs. NGN MICRO 300	-0.03417	-0.09897 to 0.03064	No	ns	>0.9999

Falls in the rotarod test

Dunn's multiple comparisons test	Mean rank diff.	Significant?	Summary	Adjusted P Value
Vehicle vs. NAR MICRO 30	6.917	No	ns	>0.9999
Vehicle vs. NAR COM 30	1.167	No	ns	>0.9999
Vehicle vs. NAR MICRO 100	13.83	No	ns	>0.9999
Vehicle vs. NAR COM 100	0.000	No	ns	>0.9999
Vehicle vs. NAR COM 300	13.83	No	ns	>0.9999
Vehicle vs. NAR Micro 300	13.83	No	ns	>0.9999
Vehicle vs. NGN COM 30	-3.417	No	ns	>0.9999
Vehicle vs. NGN MICRO 30	1.167	No	ns	>0.9999
Vehicle vs. NGN COM 100	0.000	No	ns	>0.9999
Vehicle vs. NGN MICRO 100	13.83	No	ns	>0.9999
Vehicle vs. NGN COM 300	13.83	No	ns	>0.9999
Vehicle vs. NGN MICRO 300	13.83	No	ns	>0.9999

Comparisons for TRPV1 relative expression

Outcome	Treatments	Test	F value	df	P value
**TRPV1 relative expression**	Vehicle, Naringin and Micronised naringin	One-way ANOVA	F = 7.428	(3.19)	0.0017
	Vehicle, Naringenin and Micronised naringenin	One-way ANOVA	F = 5.243	(3, 19)	0.0083

Bonferroni's multiple comparisons test	Mean Diff.	95.00% CI of diff.	Significant?	Summary	Adjusted P Value
Pacli vs. Veículo	0.001571	0.0005252 to 0.002616	Yes	**	0.0026
Pacli vs. NAR	0.0004868	-0.0005586 to 0.001532	No	ns	0.7095
Pacli vs. NAR micro	0.001459	0.0004132 to 0.002504	Yes	**	0.0050

Bonferroni's multiple comparisons test	Mean Diff.	95.00% CI of diff.	Significant?	Summary	Adjusted P Value
Pacli vs. Veículo	0.001571	0.0002702 to 0.002871	Yes	*	0.0151
Pacli vs. NGN	0.001810	0.0005099 to 0.003111	Yes	**	0.0051
Pacli vs. NGN micro	0.0009478	-0.0003526 to 0.002248	No	ns	0.2127

Comparisons for paclitaxel-induced chronic pain model - Mechanical allodynia

Outcome	Treatments	Test	F value	df	P value
**Mechanical allodynia**	Conventional and Micronised naringin	Two-way repeated measures ANOVA	Interaction: F = 2.888	(12, 84)	0.0022
			Time: F = 36.24	(3.66, 51.28)	< 0.0001
**Mechanical allodynia - AUC**	Conventional and Micronised naringin	One-Way ANOVA	F = 15.31	(2 ,14)	0.0003
**Mechanical allodynia**	Conventional and Micronised naringenin	Two-way repeated measures ANOVA	Interaction: F = 1.709	(12, 84)	0.0792
			Time: F = 28.64	(2.73, 38.28)	< 0.0001
**Mechanical allodynia - AUC**	Conventional and Micronised naringenin	One-way ANOVA	F = 32.65	(2, 14)	< 0.0001

Naringin- Mechanical allodynia - Bonferroni's multiple comparisons test

A = Paclitaxel + NAR Micro B = Paclitaxel + NAR COM C = Paclitaxel + Vehicle

	2way ANOVA Multiple comparisons					
19	0.5					
20	Group A vs. Group B	0.1740	-0.06419 to 0.4122	No	ns	0.1855
21	Group A vs. Group C	0.2096	0.01622 to 0.4031	Yes	*	0.0337
22	Group B vs. Group C	0.03563	-0.1898 to 0.2611	No	ns	>0.9999
23						
24	1					
25	Group A vs. Group B	0.2326	-0.1140 to 0.5792	No	ns	0.2430
26	Group A vs. Group C	0.4284	0.09047 to 0.7664	Yes	*	0.0196
27	Group B vs. Group C	0.1958	-0.05245 to 0.4441	No	ns	0.1157
28						
29	2					
30	Group A vs. Group B	0.2184	-0.09461 to 0.5314	No	ns	0.2158
31	Group A vs. Group C	0.5203	0.2073 to 0.8334	Yes	**	0.0028
32	Group B vs. Group C	0.3020	0.03332 to 0.5706	Yes	*	0.0278
33						
34	4					
35	Group A vs. Group B	0.3987	-0.03754 to 0.8349	No	ns	0.0748
36	Group A vs. Group C	0.4214	0.07542 to 0.7673	Yes	*	0.0184
37	Group B vs. Group C	0.02270	-0.4281 to 0.4735	No	ns	>0.9999
38						
39	6					
40	Group A vs. Group B	0.04135	-0.1952 to 0.2778	No	ns	>0.9999
41	Group A vs. Group C	0.1711	-0.04223 to 0.3844	No	ns	0.1292
42	Group B vs. Group C	0.1298	-0.1066 to 0.3661	No	ns	0.4237

Naringin- Mechanical allodynia - Basal - Bonferroni's multiple comparisons test

A = Paclitaxel + NAR Micro B = Paclitaxel + NAR COM C = Paclitaxel + Vehicle

Group A					
B1 vs. B2	2.124	0.7266 to 3.521	Yes	**	0.0082
B1 vs. 0.5	1.789	0.1832 to 3.394	Yes	*	0.0320
B1 vs. 1	0.9295	-0.1634 to 2.022	No	ns	0.0944
B1 vs. 2	0.9424	-0.2683 to 2.153	No	ns	0.1311
B1 vs. 4	1.125	-0.3415 to 2.592	No	ns	0.1382
B1 vs. 6	1.737	0.5334 to 2.941	Yes	*	0.0104
Group B					
B1 vs. B2	2.535	0.6573 to 4.413	Yes	*	0.0140
B1 vs. 0.5	2.418	0.7201 to 4.116	Yes	*	0.0110
B1 vs. 1	2.029	0.3310 to 3.727	Yes	*	0.0238
B1 vs. 2	2.015	-0.2479 to 4.278	No	ns	0.0792
B1 vs. 4	2.327	0.1170 to 4.537	Yes	*	0.0405
B1 vs. 6	2.173	0.1926 to 4.153	Yes	*	0.0341
Group C					
B1 vs. B2	2.340	0.8339 to 3.845	Yes	**	0.0100
B1 vs. 0.5	2.321	0.8403 to 3.802	Yes	**	0.0096
B1 vs. 1	2.251	0.8725 to 3.630	Yes	**	0.0082
B1 vs. 2	2.345	1.030 to 3.660	Yes	**	0.0059
B1 vs. 4	2.250	0.9589 to 3.542	Yes	**	0.0064
B1 vs. 6	2.237	0.8324 to 3.641	Yes	**	0.0091

Naringin- Mechanical allodynia - AUC - Bonferroni's multiple comparisons test

Bonferroni's multiple comparisons test	Mean Diff.	95.00% CI of diff.	Significant?	Summary	Adjusted P Value
Pacli + vehicle vs. Pacli + Nar convencional	-1.101	-2.906 to 0.7032	No	ns	0.3583
Pacli + vehicle vs. Pacli + Nar Micronized	-3.538	-5.342 to -1.733	Yes	***	0.0003
Pacli + Nar convencional vs. Pacli + Nar Micronize	-2.437	-4.157 to -0.7162	Yes	**	0.0053

Naringenin- Mechanical allodynia - Bonferroni's multiple comparisons test

A = Paclitaxel + NGN Micro B = Paclitaxel + NGN COM C = Paclitaxel + Vehicle

	2way ANOVA Multiple comparisons					
19	0.5					
20	Group A vs. Group B	0.1042	-0.1859 to 0.3942	No	ns	0.9788
21	Group A vs. Group C	0.1403	-0.09600 to 0.3767	No	ns	0.3316
22	Group B vs. Group C	0.03618	-0.2247 to 0.2971	No	ns	>0.9999
23						
24	1					
25	Group A vs. Group B	0.2083	0.03415 to 0.3824	Yes	*	0.0198
26	Group A vs. Group C	0.2861	0.1158 to 0.4563	Yes	**	0.0058
27	Group B vs. Group C	0.07780	-0.04579 to 0.2014	No	ns	0.2301
28						
29	2					
30	Group A vs. Group B	0.3064	0.01148 to 0.6014	Yes	*	0.0416
31	Group A vs. Group C	0.5884	-0.05691 to 1.234	No	ns	0.0718
32	Group B vs. Group C	0.2820	-0.3762 to 0.9401	No	ns	0.5629
33						
34	4					
35	Group A vs. Group B	0.1808	-0.4817 to 0.8434	No	ns	>0.9999
36	Group A vs. Group C	0.06011	-0.5259 to 0.6461	No	ns	>0.9999
37	Group B vs. Group C	-0.1207	-0.6687 to 0.4273	No	ns	>0.9999
38						
39	6					
40	Group A vs. Group B	0.1376	-0.1024 to 0.3776	No	ns	0.3707
41	Group A vs. Group C	0.04212	-0.1534 to 0.2376	No	ns	>0.9999
42	Group B vs. Group C	-0.09551	-0.3464 to 0.1553	No	ns	0.8711

Naringenin- Mechanical allodynia - Basal - Bonferroni's multiple comparisons test

A = Paclitaxel + NGN Micro B = Paclitaxel + NGN COM C = Paclitaxel + Vehicle

Group A					
B1 vs. B2	0.7186	0.5306 to 0.9066	Yes	***	0.0001
B1 vs. 0.5	0.5875	0.2263 to 0.9487	Yes	**	0.0060
B1 vs. 1	0.3777	0.1256 to 0.6298	Yes	**	0.0088
B1 vs. 2	0.3583	-0.06883 to 0.7855	No	ns	0.0994
B1 vs. 4	0.6394	-0.1960 to 1.475	No	ns	0.1393
B1 vs. 6	0.6223	0.2620 to 0.9826	Yes	**	0.0046
Group B					
B1 vs. B2	0.7040	0.5993 to 0.8086	Yes	****	<0.0001
B1 vs. 0.5	0.6516	0.2686 to 1.035	Yes	**	0.0049
B1 vs. 1	0.5459	0.3295 to 0.7623	Yes	***	0.0008
B1 vs. 2	0.6247	0.4816 to 0.7679	Yes	****	<0.0001
B1 vs. 4	0.7801	0.1245 to 1.436	Yes	*	0.0242
B1 vs. 6	0.7199	0.2910 to 1.149	Yes	**	0.0052
Group C					
B1 vs. B2	0.7694	0.3350 to 1.204	Yes	**	0.0061
B1 vs. 0.5	0.7475	0.3674 to 1.128	Yes	**	0.0040
B1 vs. 1	0.6834	0.4276 to 0.9393	Yes	**	0.0012
B1 vs. 2	0.9664	0.03671 to 1.896	Yes	*	0.0436
B1 vs. 4	0.7191	0.3091 to 1.129	Yes	**	0.0063
B1 vs. 6	0.6841	0.3279 to 1.040	Yes	**	0.0044

Naringenin- Mechanical allodynia - AUC - Bonferroni's multiple comparisons test

Bonferroni's multiple comparisons test	Mean Diff.	95.00% CI of diff.	Significant?	Summary	Adjusted P Value
Pacli + vehicle vs. Pacli + NGN convencional	-0.2414	-0.9085 to 0.4257	No	ns	>0.9999
Pacli + vehicle vs. Pacli + NGN Micronized	-1.782	-2.449 to -1.115	Yes	****	<0.0001
Pacli + NGN convencional vs. Pacli + NGN Micronized	-1.540	-2.176 to -0.9043	Yes	****	<0.0001

Comparisons for paclitaxel-induced chronic pain model - Cold allodynia

Outcome	Treatments	Test	F value	df	P value
**Cold allodynia**	Conventional and Micronised naringin	Mixed-effects model (REML)	F = 28.84	(3.87, 54.24)	<0.0001
**Cold allodynia - AUC**	Conventional and Micronised naringin	One-way ANOVA	F = 3.357	(2, 14)	0.064
**Cold allodynia**	Conventional and Micronised naringenin	Mixed-effects model (REML)	F = 42.06	(3.86, 54.05)	<0.0001
**Cold allodynia - AUC**	Conventional and Micronised naringenin	One-way ANOVA	F = 3.503	(2, 14)	0.058

Naringin - Cold allodynia - Bonferroni’s multiple comparisons test

A = Paclitaxel + NAR Micro B = Paclitaxel + NAR COM C = Paclitaxel + Vehicle

	Mixed-effects analysis<br>Multiple comparisons					
19	1					
20	Group A vs. Group B	-1.667	-5.087 to 1.754	No	ns	0.5589
21	Group A vs. Group C	-2.933	-5.281 to -0.5856	Yes	*	0.0165
22	Group B vs. Group C	-1.267	-4.554 to 2.020	No	ns	0.7877
23						
24	2					
25	Group A vs. Group B	-0.6667	-5.196 to 3.863	No	ns	>0.9999
26	Group A vs. Group C	-3.767	-7.369 to -0.1644	Yes	*	0.0413
27	Group B vs. Group C	-3.100	-7.156 to 0.9556	No	ns	0.1381
28						
29	4					
30	Group A vs. Group B	0.6667	-3.072 to 4.405	No	ns	>0.9999
31	Group A vs. Group C	-1.533	-5.309 to 2.243	No	ns	0.7520
32	Group B vs. Group C	-2.200	-4.361 to -0.03934	Yes	*	0.0462
33						
34	6					
35	Group A vs. Group B	-1.667	-3.978 to 0.6442	No	ns	0.1822
36	Group A vs. Group C	-1.800	-3.340 to -0.2601	Yes	*	0.0227
37	Group B vs. Group C	-0.1333	-2.462 to 2.196	No	ns	>0.9999
38						
39	8					
40	Group A vs. Group B	0.5000	-1.793 to 2.793	No	ns	>0.9999
41	Group A vs. Group C	0.2667	-2.030 to 2.563	No	ns	>0.9999
42	Group B vs. Group C	-0.2333	-1.389 to 0.9227	No	ns	>0.9999

Naringin - Cold allodynia - Basal - Bonferroni's multiple comparisons test

A = Paclitaxel + NAR Micro B = Paclitaxel + NAR COM C = Paclitaxel + Vehicle

Mixed-effects analysis<br>Multiple comparisons					
Group A					
B1 vs. B2	-4.833	-8.176 to -1.490	Yes	*	0.0103
B1 vs. 1	-1.833	-4.994 to 1.327	No	ns	0.3487
B1 vs. 2	-1.000	-5.358 to 3.358	No	ns	>0.9999
B1 vs. 4	-2.833	-7.111 to 1.444	No	ns	0.2294
B1 vs. 6	-2.167	-4.702 to 0.3688	No	ns	0.0927
B1 vs. 8	-3.833	-6.369 to -1.298	Yes	**	0.0084
Group B					
B1 vs. B2	-5.833	-10.11 to -1.556	Yes	*	0.0133
B1 vs. 1	-4.000	-8.076 to 0.07624	No	ns	0.0540
B1 vs. 2	-2.167	-7.871 to 3.538	No	ns	>0.9999
B1 vs. 4	-2.667	-5.874 to 0.5405	No	ns	0.1028
B1 vs. 6	-4.333	-7.720 to -0.9462	Yes	*	0.0177
B1 vs. 8	-3.833	-5.847 to -1.820	Yes	**	0.0029
Group C					
B1 vs. B2	-6.200	-8.576 to -3.824	Yes	**	0.0013
B1 vs. 1	-6.000	-7.534 to -4.466	Yes	***	0.0003
B1 vs. 2	-6.000	-7.534 to -4.466	Yes	***	0.0003
B1 vs. 4	-5.600	-8.074 to -3.126	Yes	**	0.0023
B1 vs. 6	-5.200	-7.576 to -2.824	Yes	**	0.0027
B1 vs. 8	-4.800	-7.176 to -2.424	Yes	**	0.0036

Naringin- Cold allodynia - AUC - Bonferroni's multiple comparisons test

Bonferroni's multiple comparisons test	Mean Diff.	95.00% CI of diff.	Significant?	Summary	Adjusted P Value
Pacli + vehicle vs. Pacli + Nar convencional	6.183	-2.881 to 15.25	No	ns	0.2548
Pacli + vehicle vs. Pacli + Nar Micronized	8.433	-0.6312 to 17.50	No	ns	0.0723
Pacli + Nar convencional vs. Pacli + Nar Micronized	2.250	-6.393 to 10.89	No	ns	>0.9999

Naringenin - Cold allodynia - Bonferroni's multiple comparisons test

A = Paclitaxel + NGN Micro B = Paclitaxel + NGN COM C = Paclitaxel + Vehicle

Mixed-effects analysis<br>Multiple comparisons					
1					
Group A vs. Group B	-0.5000	-2.430 to 1.430	No	ns	>0.9999
Group A vs. Group C	-2.600	-4.360 to -0.8400	Yes	**	0.0057
Group B vs. Group C	-2.100	-3.983 to -0.2168	Yes	*	0.0291
2					
Group A vs. Group B	-1.000	-4.446 to 2.446	No	ns	>0.9999
Group A vs. Group C	-2.933	-5.574 to -0.2931	Yes	*	0.0306
Group B vs. Group C	-1.933	-5.099 to 1.233	No	ns	0.2894
4					
Group A vs. Group B	0.5000	-1.568 to 2.568	No	ns	>0.9999
Group A vs. Group C	-0.3667	-2.826 to 2.093	No	ns	>0.9999
Group B vs. Group C	-0.8667	-3.049 to 1.316	No	ns	0.6616
6					
Group A vs. Group B	0.6667	-2.153 to 3.486	No	ns	>0.9999
Group A vs. Group C	-0.8000	-3.616 to 2.016	No	ns	>0.9999
Group B vs. Group C	-1.467	-3.120 to 0.1869	No	ns	0.0860
8					
Group A vs. Group B	0.1667	-2.551 to 2.884	No	ns	>0.9999
Group A vs. Group C	-0.4000	-3.075 to 2.275	No	ns	>0.9999
Group B vs. Group C	-0.5667	-2.222 to 1.088	No	ns	0.9730

Naringenin - Cold allodynia - Basal - Bonferroni's multiple comparisons test

A = Paclitaxel + NGN Micro B = Paclitaxel + NGN COM C = Paclitaxel + Vehicle

Mixed-effects analysis<br>Multiple comparisons					
Group A					
B1 vs. B2	-5.500	-7.874 to -3.126	Yes	**	0.0011
B1 vs. 1	-3.000	-6.928 to 0.9280	No	ns	0.1404
B1 vs. 2	-2.667	-7.967 to 2.633	No	ns	0.5231
B1 vs. 4	-4.833	-8.824 to -0.8429	Yes	*	0.0224
B1 vs. 6	-4.000	-8.622 to 0.6220	No	ns	0.0884
B1 vs. 8	-4.000	-8.872 to 0.8720	No	ns	0.1078
Group B					
B1 vs. B2	-5.833	-8.123 to -3.544	Yes	***	0.0007
B1 vs. 1	-4.000	-7.445 to -0.5549	Yes	*	0.0269
B1 vs. 2	-4.167	-8.444 to 0.1108	No	ns	0.0556
B1 vs. 4	-4.833	-6.527 to -3.140	Yes	***	0.0004
B1 vs. 6	-3.833	-6.593 to -1.074	Yes	*	0.0123
B1 vs. 8	-4.333	-6.687 to -1.980	Yes	**	0.0034
Group C					
B1 vs. B2	-6.200	-8.576 to -3.824	Yes	**	0.0013
B1 vs. 1	-6.000	-7.534 to -4.466	Yes	***	0.0003
B1 vs. 2	-6.000	-7.534 to -4.466	Yes	***	0.0003
B1 vs. 4	-5.600	-8.074 to -3.126	Yes	**	0.0023
B1 vs. 6	-5.200	-7.576 to -2.824	Yes	**	0.0027
B1 vs. 8	-4.800	-7.176 to -2.424	Yes	**	0.0036

Naringenin- Cold allodynia - AUC - Bonferroni's multiple comparisons test

Bonferroni's multiple comparisons test	Mean Diff.	95.00% CI of diff.	Significant?	Summary	Adjusted P Value
Pacli + vehicle vs. Pacli + NGN convencional	5.600	-0.9415 to 12.14	No	ns	0.1065
Pacli + vehicle vs. Pacli + NGN Micronized	5.600	-0.9415 to 12.14	No	ns	0.1065
Pacli + NGN convencional vs. Pacli + NGN Micronized	0.000	-6.237 to 6.237	No	ns	>0.9999

Comparisons for paclitaxel-induced acute pain model - Mechanical allodynia

Outcome	Treatments	Test	F value	df	P value
**Mechanical allodynia**	Conventional and Micronised naringin	Two-way repeated measures ANOVA	Interaction F = 2.803	(14, 105)	P=0.0014
			Time F=59.05	(3.82, 57.05)	P<0.0001
**Mechanical allodynia - AUC**	Conventional and Micronised naringin	One-way ANOVA	F = 15.45	(2, 15)	P= 0.0002
**Mechanical allodynia**	Conventional and Micronised naringenin	Two-way repeated measures ANOVA	Interaction F = 3.238	(14, 105)	P = 0.0003
			Time F= 45.30	(4.68, 70.24)	P<0.0001
**Mechanical allodynia - AUC**	Conventional and Micronised naringenin	One-way ANOVA	F = 23.44	(2, 15)	P<0.0001

Naringin- Mechanical allodynia - Bonferroni's multiple comparisons test

A = Paclitaxel + Vehicle B = Paclitaxel + NAR COM C = Paclitaxel + NAR Micro

0.5					
Group A vs. Group B	-0.06276	-0.4322 to 0.3067	No	ns	>0.9999
Group A vs. Group C	-0.2368	-0.5797 to 0.1062	No	ns	0.1938
Group B vs. Group C	-0.1740	-0.4474 to 0.09930	No	ns	0.2582
1					
Group A vs. Group B	-0.1062	-0.2416 to 0.02918	No	ns	0.1442
Group A vs. Group C	-0.3184	-0.4961 to -0.1407	Yes	**	0.0016
Group B vs. Group C	-0.2122	-0.3894 to -0.03505	Yes	*	0.0200
2					
Group A vs. Group B	-0.1321	-0.3810 to 0.1168	No	ns	0.4685
Group A vs. Group C	-0.5097	-0.8280 to -0.1914	Yes	**	0.0032
Group B vs. Group C	-0.3776	-0.6800 to -0.07518	Yes	*	0.0163
4					
Group A vs. Group B	-0.2274	-0.5023 to 0.04744	No	ns	0.1125
Group A vs. Group C	-0.4626	-0.7411 to -0.1842	Yes	**	0.0028
Group B vs. Group C	-0.2352	-0.4390 to -0.03137	Yes	*	0.0236
6					
Group A vs. Group B	-0.001553	-0.2784 to 0.2753	No	ns	>0.9999
Group A vs. Group C	-0.1575	-0.3712 to 0.05621	No	ns	0.1815
Group B vs. Group C	-0.1560	-0.4329 to 0.1210	No	ns	0.3963
8					
Group A vs. Group B	0.01730	-0.2822 to 0.3168	No	ns	>0.9999
Group A vs. Group C	-0.08734	-0.3269 to 0.1522	No	ns	0.9545
Group B vs. Group C	-0.1046	-0.4140 to 0.2047	No	ns	>0.9999

Naringin- Mechanical allodynia - Basal - Bonferroni's multiple comparisons test

A = Paclitaxel + Vehicle B = Paclitaxel + NAR COM C = Paclitaxel + NAR Micro

Group A					
B1 vs. B2	0.6995	0.4821 to 0.9169	Yes	****	<0.0001
B1 vs. 0.5	0.7260	0.5086 to 0.9434	Yes	****	<0.0001
B1 vs. 1	0.6218	0.4044 to 0.8392	Yes	****	<0.0001
B1 vs. 2	0.6477	0.4303 to 0.8651	Yes	****	<0.0001
B1 vs. 4	0.7694	0.5521 to 0.9868	Yes	****	<0.0001
B1 vs. 6	0.6736	0.4562 to 0.8910	Yes	****	<0.0001
B1 vs. 8	0.6812	0.4638 to 0.8986	Yes	****	<0.0001
Group B					
B1 vs. B2	0.7837	0.5663 to 1.001	Yes	****	<0.0001
B1 vs. 0.5	0.7473	0.5299 to 0.9647	Yes	****	<0.0001
B1 vs. 1	0.5997	0.3823 to 0.8171	Yes	****	<0.0001
B1 vs. 2	0.5998	0.3824 to 0.8171	Yes	****	<0.0001
B1 vs. 4	0.6262	0.4088 to 0.8435	Yes	****	<0.0001
B1 vs. 6	0.7562	0.5388 to 0.9736	Yes	****	<0.0001
B1 vs. 8	0.7826	0.5652 to 1.000	Yes	****	<0.0001
Group C					
B1 vs. B2	0.8423	0.6249 to 1.060	Yes	****	<0.0001
B1 vs. 0.5	0.6304	0.4130 to 0.8478	Yes	****	<0.0001
B1 vs. 1	0.4446	0.2272 to 0.6620	Yes	****	<0.0001
B1 vs. 2	0.2793	0.06188 to 0.4967	Yes	**	0.0044
B1 vs. 4	0.4481	0.2307 to 0.6655	Yes	****	<0.0001
B1 vs. 6	0.6574	0.4400 to 0.8748	Yes	****	<0.0001
B1 vs. 8	0.7351	0.5177 to 0.9525	Yes	****	<0.0001

Naringin- Mechanical allodynia - AUC - Bonferroni's multiple comparisons test

Bonferroni's multiple comparisons test	Mean Diff.	95.00% CI of diff.	Significant?	Summary	Adjusted P Value
Pacli + Vehi vs. Nar CON	-0.5872	-2.025 to 0.8508	No	ns	0.8661
Pacli + Vehi vs. Nar MICRO	-2.812	-4.250 to -1.374	Yes	***	0.0003
Nar CON vs. Nar MICRO	-2.225	-3.663 to -0.7872	Yes	**	0.0025

Naringenin- Mechanical allodynia - Bonferroni's multiple comparisons test

A = Paclitaxel + Vehicle B = Paclitaxel + NGN COM C = Paclitaxel + NGN Micro

0.5					
Group A vs. Group B	-0.2627	-0.6070 to 0.08158	No	ns	0.1486
Group A vs. Group C	-0.3668	-0.7102 to -0.02337	Yes	*	0.0379
Group B vs. Group C	-0.1041	-0.2871 to 0.07891	No	ns	0.3805
1					
Group A vs. Group B	-0.1844	-0.3566 to -0.01222	Yes	*	0.0357
Group A vs. Group C	-0.4520	-0.7208 to -0.1832	Yes	**	0.0037
Group B vs. Group C	-0.2676	-0.5419 to 0.006750	No	ns	0.0561
2					
Group A vs. Group B	-0.1590	-0.3926 to 0.07450	No	ns	0.2171
Group A vs. Group C	-0.3404	-0.5733 to -0.1074	Yes	**	0.0075
Group B vs. Group C	-0.1813	-0.3185 to -0.04414	Yes	*	0.0106
4					
Group A vs. Group B	-0.2803	-0.5498 to -0.01074	Yes	*	0.0421
Group A vs. Group C	-0.4138	-0.7081 to -0.1194	Yes	**	0.0073
Group B vs. Group C	-0.1335	-0.3586 to 0.09163	No	ns	0.3295
6					
Group A vs. Group B	-0.1062	-0.2992 to 0.08676	No	ns	0.4282
Group A vs. Group C	-0.2771	-0.5226 to -0.03157	Yes	*	0.0269
Group B vs. Group C	-0.1709	-0.4036 to 0.06174	No	ns	0.1745
8					
Group A vs. Group B	-0.1137	-0.3098 to 0.08231	No	ns	0.3727
Group A vs. Group C	0.05972	-0.3053 to 0.4247	No	ns	>0.9999
Group B vs. Group C	0.1735	-0.1901 to 0.5371	No	ns	0.5198

Naringenin- Mechanical allodynia - Basal - Bonferroni's multiple comparisons test

A = Paclitaxel + Vehicle B = Paclitaxel + NGN COM C = Paclitaxel + NGN Micro

B1 vs. B2	0.6995	0.4674 to 0.9317	Yes	****	<0.0001
B1 vs. 0.5	0.7260	0.4938 to 0.9581	Yes	****	<0.0001
B1 vs. 1	0.6218	0.3896 to 0.8539	Yes	****	<0.0001
B1 vs. 2	0.6477	0.4156 to 0.8798	Yes	****	<0.0001
B1 vs. 4	0.7694	0.5373 to 1.002	Yes	****	<0.0001
B1 vs. 6	0.6736	0.4415 to 0.9058	Yes	****	<0.0001
B1 vs. 8	0.6812	0.4491 to 0.9133	Yes	****	<0.0001
Group B					
B1 vs. B2	0.7445	0.5123 to 0.9766	Yes	****	<0.0001
B1 vs. 0.5	0.5336	0.3015 to 0.7658	Yes	****	<0.0001
B1 vs. 1	0.5077	0.2756 to 0.7398	Yes	****	<0.0001
B1 vs. 2	0.5590	0.3269 to 0.7912	Yes	****	<0.0001
B1 vs. 4	0.5595	0.3274 to 0.7917	Yes	****	<0.0001
B1 vs. 6	0.6378	0.4057 to 0.8699	Yes	****	<0.0001
B1 vs. 8	0.6378	0.4057 to 0.8699	Yes	****	<0.0001
Group C					
B1 vs. B2	0.8105	0.5784 to 1.043	Yes	****	<0.0001
B1 vs. 0.5	0.4433	0.2112 to 0.6754	Yes	****	<0.0001
B1 vs. 1	0.2539	0.02180 to 0.4861	Yes	*	0.0235
B1 vs. 2	0.3915	0.1594 to 0.6236	Yes	****	<0.0001
B1 vs. 4	0.4398	0.2077 to 0.6719	Yes	****	<0.0001
B1 vs. 6	0.4807	0.2485 to 0.7128	Yes	****	<0.0001
B1 vs. 8	0.8250	0.5929 to 1.057	Yes	****	<0.0001

Naringenin- Mechanical allodynia - AUC - Bonferroni's multiple comparisons test

Bonferroni's multiple comparisons test	Mean Diff.	95.00% CI of diff.	Significant?	Summary	Adjusted P Value
Pacli + Vehi vs. NGN CON	-1.123	-2.248 to 0.002154	No	ns	0.0505
Pacli + Vehi vs. NGN MICRO	-2.839	-3.964 to -1.714	Yes	****	<0.0001
NGN CON vs. NGN MICRO	-1.716	-2.841 to -0.5912	Yes	**	0.0028

Comparisons for paclitaxel-induced acute pain model - Cold allodynia

Outcome	Treatments	Test	F value	df	P value
**Cold allodynia**	Conventional and Micronised naringin	Mixed-effects model (REML)	F = 40.96	(3.44, 51.60)	<0.0001
**Cold allodynia - AUC**	Conventional and Micronised naringin	One-way ANOVA	F = 10.56	(2, 15)	0.0014
**Cold allodynia**	Conventional and Micronised naringenin	Mixed-effects model (REML)	F = 32.00	(4.78, 71.81)	<0.0001
**Cold allodynia - AUC**	Conventional and Micronised naringenin	One-way ANOVA	F = 11.76	(2, 15)	0.0008

Naringin – Cold allodynia Bonferroni's multiple comparisons test

A = Paclitaxel + Vehicle B = Paclitaxel + NAR COM C = Paclitaxel + NAR Micro

Mixed-effects analysis<br>Multiple comparisons					
1					
Group A vs. Group B	2.500	0.2073 to 4.793	Yes	*	0.0338
Group A vs. Group C	3.500	1.044 to 5.956	Yes	**	0.0091
Group B vs. Group C	1.000	-1.808 to 3.808	No	ns	0.9916
2					
Group A vs. Group B	1.667	-0.3382 to 3.672	No	ns	0.1136
Group A vs. Group C	2.833	0.4805 to 5.186	Yes	*	0.0195
Group B vs. Group C	1.167	-1.320 to 3.654	No	ns	0.6172
4					
Group A vs. Group B	1.333	-0.6772 to 3.344	No	ns	0.2415
Group A vs. Group C	2.333	-0.4720 to 5.139	No	ns	0.1046
Group B vs. Group C	1.000	-1.933 to 3.933	No	ns	>0.9999
6					
Group A vs. Group B	0.6667	-1.520 to 2.853	No	ns	>0.9999
Group A vs. Group C	1.833	0.5304 to 3.136	Yes	**	0.0071
Group B vs. Group C	1.167	-1.012 to 3.345	No	ns	0.4189
8					
Group A vs. Group B	0.1667	-1.211 to 1.544	No	ns	>0.9999
Group A vs. Group C	0.6667	-0.4446 to 1.778	No	ns	0.3384
Group B vs. Group C	0.5000	-0.7801 to 1.780	No	ns	0.8262

Naringin – Cold allodynia - Basal - Bonferroni's multiple comparisons test

A = Paclitaxel + Vehicle B = Paclitaxel + NAR COM C = Paclitaxel + NAR Micro

Mixed-effects analysis<br>Multiple comparisons					
**Bonferroni's multiple comparisons**	**Mean Diff.**	**95.00% CI of diff.**	**Significant?**	**Summary**	**Adjusted P Value**
Group A					
B1 vs. B2	-4.500	-6.610 to -2.390	Yes	**	0.0017
B1 vs. 1	-4.333	-6.112 to -2.554	Yes	***	0.0009
B1 vs. 2	-4.667	-6.446 to -2.888	Yes	***	0.0006
B1 vs. 4	-4.500	-5.941 to -3.059	Yes	***	0.0003
B1 vs. 6	-4.833	-7.123 to -2.544	Yes	**	0.0018
B1 vs. 8	-4.333	-6.112 to -2.554	Yes	***	0.0009
Group B					
B1 vs. B2	-5.000	-7.882 to -2.118	Yes	**	0.0045
B1 vs. 1	-1.333	-5.211 to 2.544	No	ns	>0.9999
B1 vs. 2	-2.500	-6.072 to 1.072	No	ns	0.1906
B1 vs. 4	-2.667	-6.544 to 1.211	No	ns	0.2023
B1 vs. 6	-3.667	-6.020 to -1.313	Yes	**	0.0073
B1 vs. 8	-3.667	-6.480 to -0.8538	Yes	*	0.0163
Group C					
B1 vs. B2	-5.333	-6.740 to -3.927	Yes	***	0.0001
B1 vs. 1	-1.167	-4.510 to 2.176	No	ns	>0.9999
B1 vs. 2	-2.167	-5.510 to 1.176	No	ns	0.2463
B1 vs. 4	-2.500	-5.723 to 0.7226	No	ns	0.1327
B1 vs. 6	-3.333	-4.223 to -2.444	Yes	***	0.0001
B1 vs. 8	-4.000		Yes	****	<0.0001

Naringin – Cold allodynia - AUC - Bonferroni's multiple comparisons test

Bonferroni's multiple comparisons test	Mean Diff.	95.00% CI of diff.	Significant?	Summary	Adjusted P Value
Pacli + Vehi vs. Nar CON	5.000	-0.3330 to 10.33	No	ns	0.0699
Pacli + Vehi vs. Nar MICRO	9.083	3.750 to 14.42	Yes	**	0.0011
Nar CON vs. Nar MICRO	4.083	-1.250 to 9.416	No	ns	0.1708

Naringenin – Cold allodynia   Bonferroni's multiple comparisons test

A = Paclitaxel + Vehicle   B = Paclitaxel + NGN COM   C = Paclitaxel + NGN Micro

1					
Group A vs. Group B	0.5000	-0.8029 to 1.803	No	ns	0.8883
Group A vs. Group C	2.500	0.9748 to 4.025	Yes	**	0.0028
Group B vs. Group C	2.000	0.4412 to 3.559	Yes	*	0.0130
2					
Group A vs. Group B	1.000	-1.214 to 3.214	No	ns	0.8514
Group A vs. Group C	2.000	-0.05788 to 4.058	No	ns	0.0573
Group B vs. Group C	1.000	-1.406 to 3.406	No	ns	0.7794
4					
Group A vs. Group B	1.333	-0.08795 to 2.755	No	ns	0.0678
Group A vs. Group C	1.500	-0.2101 to 3.210	No	ns	0.0900
Group B vs. Group C	0.1667	-1.581 to 1.915	No	ns	>0.9999
6					
Group A vs. Group B	1.667	0.4417 to 2.892	Yes	**	0.0092
Group A vs. Group C	1.500	-0.5851 to 3.585	No	ns	0.1842
Group B vs. Group C	-0.1667	-2.231 to 1.898	No	ns	>0.9999
8					
Group A vs. Group B	0.6667	-0.4446 to 1.778	No	ns	0.3384
Group A vs. Group C	0.8333	-1.101 to 2.768	No	ns	0.6840
Group B vs. Group C	0.1667	-1.750 to 2.083	No	ns	>0.9999

Naringenin – Cold allodynia – Basal - Bonferroni's multiple comparisons test

A = Paclitaxel + Vehicle   B = Paclitaxel + NGN COM   C = Paclitaxel + NGN Micro

Group A					
B1 vs. B2	-4.500	-6.610 to -2.390	Yes	**	0.0017
B1 vs. 1	-4.333	-6.112 to -2.554	Yes	***	0.0009
B1 vs. 2	-4.667	-6.446 to -2.888	Yes	***	0.0006
B1 vs. 4	-4.500	-5.941 to -3.059	Yes	***	0.0003
B1 vs. 6	-4.833	-7.123 to -2.544	Yes	**	0.0018
B1 vs. 8	-4.333	-6.112 to -2.554	Yes	***	0.0009
Group B					
B1 vs. B2	-3.500	-5.874 to -1.126	Yes	**	0.0094
B1 vs. 1	-3.000	-4.887 to -1.113	Yes	**	0.0067
B1 vs. 2	-2.833	-4.847 to -0.8196	Yes	*	0.0116
B1 vs. 4	-2.333	-4.419 to -0.2473	Yes	*	0.0315
B1 vs. 6	-2.333	-4.927 to 0.2600	No	ns	0.0761
B1 vs. 8	-2.833	-4.847 to -0.8196	Yes	*	0.0116
Group C					
B1 vs. B2	-3.833	-6.369 to -1.298	Yes	**	0.0084
B1 vs. 1	-1.500	-4.723 to 1.723	No	ns	0.6405
B1 vs. 2	-2.333	-4.687 to 0.02008	No	ns	0.0518
B1 vs. 4	-2.667	-5.683 to 0.3498	No	ns	0.0814
B1 vs. 6	-3.000	-5.882 to -0.1177	Yes	*	0.0425
B1 vs. 8	-3.167	-6.134 to -0.1998	Yes	*	0.0383

Naringenin – Cold allodynia – AUC - Bonferroni's multiple comparisons test

Bonferroni's multiple comparisons test	Mean Diff.	95.00% CI of diff.	Significant?	Summary	Adjusted P Value
Pacli + Vehi vs. NGN CON	4.583	0.7941 to 8.373	Yes	*	0.0159
Pacli + Vehi vs. NGN MICRO	6.667	2.877 to 10.46	Yes	***	0.0008
NGN CON vs. NGN MICRO	2.083	-1.706 to 5.873	No	ns	0.4779

Comparisons for paclitaxel-induced heat hyperalgesia

Outcome	Formulation	Test	Statistic	df	P value
**Thermal hyperalgesia (acute model)**	Conventional and Micronised naringin and naringenin	One-way ANOVA	F = 28.30	(5, 28)	< 0.0001
**Thermal hyperalgesia (chronic model)**	Conventional and Micronised naringin and naringenin	One-way ANOVA	F = 5.003	(5, 30)	0.0019

Acute:

Bonferroni's multiple comparisons test	Mean Diff.	95.00% CI of diff.	Significant?	Summary	Adjusted P Value
Vehicle + Vehicle vs. Paclitaxel + Vehicle	15.67	9.121 to 22.21	Yes	****	<0.0001
Vehicle + Vehicle vs. Paclitaxel + NAR CON	12.67	6.121 to 19.21	Yes	****	<0.0001
Vehicle + Vehicle vs. Paclitaxel + NAR MICRO	-2.467	-9.332 to 4.399	No	ns	>0.9999
Vehicle + Vehicle vs. Paclitaxel + NGN CON	13.33	6.468 to 20.20	Yes	****	<0.0001
Vehicle + Vehicle vs. Paclitaxel + NGN MICRO	1.500	-5.046 to 8.046	No	ns	>0.9999
Paclitaxel + Vehicle vs. Paclitaxel + NAR CON	-3.000	-9.546 to 3.546	No	ns	>0.9999
Paclitaxel + Vehicle vs. Paclitaxel + NAR MICRO	-18.13	-25.00 to -11.27	Yes	****	<0.0001
Paclitaxel + Vehicle vs. Paclitaxel + NGN CON	-2.333	-9.199 to 4.532	No	ns	>0.9999
Paclitaxel + Vehicle vs. Paclitaxel + NGN MICRO	-14.17	-20.71 to -7.621	Yes	****	<0.0001
Paclitaxel + NAR CON vs. Paclitaxel + NAR MICRO	-15.13	-22.00 to -8.268	Yes	****	<0.0001
Paclitaxel + NAR CON vs. Paclitaxel + NGN CON	0.6667	-6.199 to 7.532	No	ns	>0.9999
Paclitaxel + NAR CON vs. Paclitaxel + NGN MICRO	-11.17	-17.71 to -4.621	Yes	***	0.0001
Paclitaxel + NAR MICRO vs. Paclitaxel + NGN CON	15.80	8.630 to 22.97	Yes	****	<0.0001
Paclitaxel + NAR MICRO vs. Paclitaxel + NGN MICRO	3.967	-2.899 to 10.83	No	ns	>0.9999
Paclitaxel + NGN CON vs. Paclitaxel + NGN MICRO	-11.83	-18.70 to -4.968	Yes	****	<0.0001

Chronic:

Bonferroni's multiple comparisons test	Mean Diff.	95.00% CI of diff.	Significant?	Summary	Adjusted P Value
Vehicle + Vehicle vs. Paclitaxel + Vehicle	11.83	2.124 to 21.54	Yes	**	0.0078
Vehicle + Vehicle vs. Paclitaxel + NAR CON	6.000	-3.709 to 15.71	No	ns	0.8708
Vehicle + Vehicle vs. Paclitaxel + NAR MICRO	1.667	-8.042 to 11.38	No	ns	>0.9999
Vehicle + Vehicle vs. Paclitaxel + NGN CON	11.17	1.458 to 20.88	Yes	*	0.0142
Vehicle + Vehicle vs. Paclitaxel + NGN MICRO	5.333	-4.376 to 15.04	No	ns	>0.9999
Paclitaxel + Vehicle vs. Paclitaxel + NAR CON	-5.833	-15.54 to 3.876	No	ns	0.9743
Paclitaxel + Vehicle vs. Paclitaxel + NAR MICRO	-10.17	-19.88 to -0.4576	Yes	*	0.0339
Paclitaxel + Vehicle vs. Paclitaxel + NGN CON	-0.6667	-10.38 to 9.042	No	ns	>0.9999
Paclitaxel + Vehicle vs. Paclitaxel + NGN MICRO	-6.500	-16.21 to 3.209	No	ns	0.6159
Paclitaxel + NAR CON vs. Paclitaxel + NAR MICRO	-4.333	-14.04 to 5.376	No	ns	>0.9999
Paclitaxel + NAR CON vs. Paclitaxel + NGN CON	5.167	-4.542 to 14.88	No	ns	>0.9999
Paclitaxel + NAR CON vs. Paclitaxel + NGN MICRO	-0.6667	-10.38 to 9.042	No	ns	>0.9999
Paclitaxel + NAR MICRO vs. Paclitaxel + NGN CON	9.500	-0.2090 to 19.21	No	ns	0.0596
Paclitaxel + NAR MICRO vs. Paclitaxel + NGN MICRO	3.667	-6.042 to 13.38	No	ns	>0.9999
Paclitaxel + NGN CON vs. Paclitaxel + NGN MICRO	-5.833	-15.54 to 3.876	No	ns	0.9743
