# Supplementary material for: Supercritical CO2 Antisolvent-Micronised Naringin and Naringenin Alleviate Paclitaxel-Induced Pain Syndrome
Source: Pharmaceutics. 2026 Jun 17;18(6):747. doi: 10.3390/pharmaceutics18060747 (PMC13307101; doi:10.3390/pharmaceutics18060747)
Supplement: Supplementary file 1 [file pharmaceutics-18-00747-s001.zip › Supplementary Materials.pdf]

SUPLEMENTARY MATERIAL

2.4. Evaluation of the Dissolution Rate

Quantification was performed by UV–Vis spectrophotometry using external calibration curves prepared in ethanol prior to the dissolution assays. For naringenin, the calibration curve was constructed in the concentration range of 2–40 μg·mL^{-1}, showing linear behaviour with R^2 = 0.995. For naringin, the calibration curve was prepared in the range of 2–80 μg·mL^{-1}, with R^2 = 0.9992. Complete analytical validation in the dissolution media (pH 1.2 and pH 6.8) was not performed due to the low aqueous solubility of the compounds and the inherent limitations associated with direct UV–Vis analysis in aqueous media. Therefore, possible matrix effects, medium-specific interferences, and baseline contributions from PBS and HCl media cannot be completely excluded, particularly considering the complex dispersion behaviour of flavonoids under non-sink conditions. Since the dissolution assays were primarily designed for comparative evaluation between conventional and micronised particles under identical experimental conditions, the obtained profiles should be interpreted as apparent dissolution behaviour rather than absolute solubility determination or true sink dissolution kinetics. The adopted analytical approach was therefore considered suitable for comparative purposes, as all formulations were analysed under the same experimental conditions and using the same analytical procedure.

The dissolution assays were conducted under non-sink and supersaturated conditions using an excess amount of solid material. Under these conditions, the withdrawal of 2 mL aliquots without replacement represented a relatively small fraction of the total dissolution medium volume and was not expected to substantially alter the comparative behaviour of the systems throughout the experiment. Therefore, no cumulative volume correction was applied, and the results are presented as apparent concentration profiles obtained under non-replacement sampling conditions. Some measurements obtained for micronised naringin at later sampling times slightly exceeded the upper limit of the analytical calibration range (80 μg·mL^{-1}). However, these values remained close to the validated linear interval and were maintained for comparative interpretation of the apparent dissolution profiles.”

2.9. mRNA TRPV1 Expression in Paclitaxel-Induced Pain

The RefFinder program identified the combined expression of β-actin and GAPDH as more stable than either gene alone; therefore, the average of the two genes was used as a control for calculating TRPV1 expression (Figure S1).

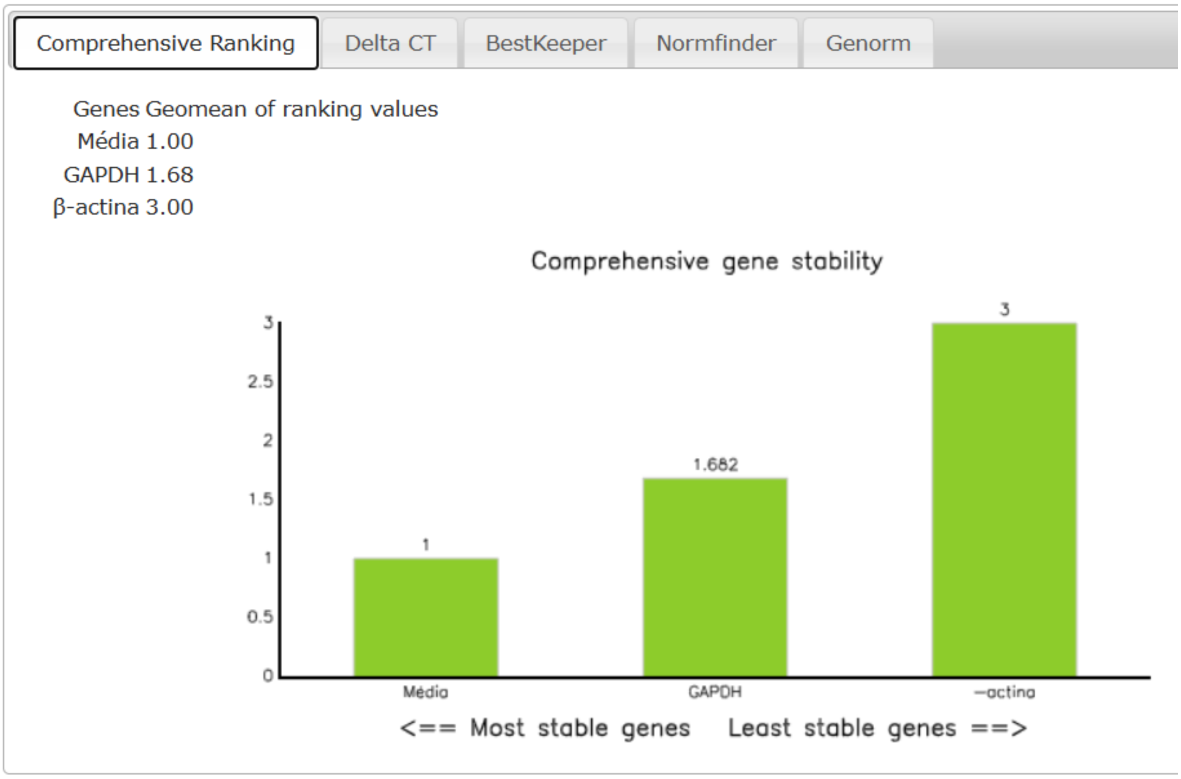

Figure S1. Gene stability classification
